# Amphiphilic Aminoglycosides as Medicinal Agents

**DOI:** 10.3390/ijms21197411

**Published:** 2020-10-08

**Authors:** Clément Dezanet, Julie Kempf, Marie-Paule Mingeot-Leclercq, Jean-Luc Décout

**Affiliations:** 1 Molecular Pharmacochemistry Department, University Grenoble Alpes, CNRS, 470 Rue de la Chimie, F-38000 Grenoble, France; clement.dezanet@gmail.com (C.D.); julie.kempf@hotmail.fr (J.K.); 2 Cellular and Molecular Pharmacology Unit, Louvain Drug Research Institute, Catholic University of Louvain, Avenue E. Mounier 73, UCL B1.73.05, 1200 Brussels, Belgium

**Keywords:** aminoglycosides, amphiphilic, antibacterial, antibiotic, cardiolipin, delivery vehicles, lipopolysaccharides, membranes

## Abstract

The conjugation of hydrophobic group(s) to the polycationic hydrophilic core of the antibiotic drugs aminoglycosides (AGs), targeting ribosomal RNA, has led to the development of amphiphilic aminoglycosides (AAGs). These drugs exhibit numerous biological effects, including good antibacterial effects against susceptible and multidrug-resistant bacteria due to the targeting of bacterial membranes. In the first part of this review, we summarize our work in identifying and developing broad-spectrum antibacterial AAGs that constitute a new class of antibiotic agents acting on bacterial membranes. The target-shift strongly improves antibiotic activity against bacterial strains that are resistant to the parent AG drugs and to antibiotic drugs of other classes, and renders the emergence of resistant *Pseudomonas aeruginosa* strains highly difficult. Structure–activity and structure–eukaryotic cytotoxicity relationships, specificity and barriers that need to be crossed in their development as antibacterial agents are delineated, with a focus on their targets in membranes, lipopolysaccharides (LPS) and cardiolipin (CL), and the corresponding mode of action against Gram-negative bacteria. At the end of the first part, we summarize the other recent advances in the field of antibacterial AAGs, mainly published since 2016, with an emphasis on the emerging AAGs which are made of an AG core conjugated to an adjuvant or an antibiotic drug of another class (antibiotic hybrids). In the second part, we briefly illustrate other biological and biochemical effects of AAGs, i.e., their antifungal activity, their use as delivery vehicles of nucleic acids, of short peptide (polyamide) nucleic acids (PNAs) and of drugs, as well as their ability to cleave DNA at abasic sites and to inhibit the functioning of connexin hemichannels. Finally, we discuss some aspects of structure–activity relationships in order to explain and improve the target selectivity of AAGs.

## 1. Introduction

**Figure 1:**
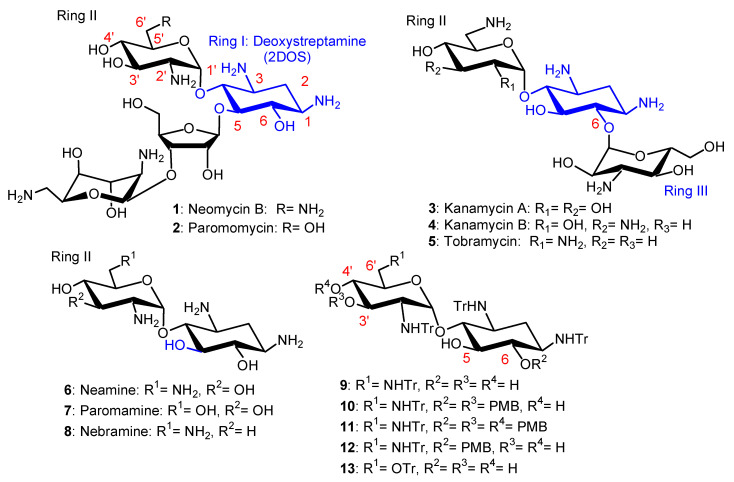
Structures of natural antibiotic aminoglycosides **1**–**5**, of some corresponding constitutive derivatives **6**–**8** and of synthetic intermediates used to prepare amphiphilic aminoglycosides (AAGs) **9**–**13** (Tr = trityl group = triphenylmethyl, PMB = *para*-methoxyphenyl group).

Aminoglycosides (AGs), for example, neomycin B (NEO) **1**, paromomycin (PARO) **2**, kanamycins A and B (KANA, KANB) **3**, **4** and tobramycin **5** (Figure 1), constitute a large family of highly potent broad-spectrum antibiotic drugs. These naturally occurring hydrophilic pseudo-oligosaccharides, which are polycationic species at physiological pH, target ribosomal RNA and disrupt protein synthesis [1]. Unfortunately, the widespread clinical use of AGs has strongly reduced their clinical efficacy through the selection of resistant bacteria [2]. In the search for new antibacterial agents targeting bacterial membranes, our team and others have synthesized and identified antibacterial amphiphilic aminoglycosides (antibacterial AAGs), which constitute a new class of polycationic antibiotic agents [3,4,5,6,7,8]. AAGs can be defined as substances made of a hydrophilic AG core on which lipophilic/hydrophobic group(s) are grafted, to produce, at physiological pH, cationic amphiphilic species.

Originally, AAGs were developed in the search for new AGs that are less susceptible to resistance. In this approach, Hanessian, Westhof and coworkers reported the first example of in vivo antibacterial lipophilic ether-modified derivatives of PARO targeting ribosomal RNA with a novel mode of binding, which are able to inhibit AG-deactivating enzymes [9,10].

In the never-ceasing fight against pathogenic multidrug-resistant bacteria, the identification of new targets, and of the corresponding drugs, is imperative [11], and an interest in membrane-targeting drug is emerging [12]. The expected major advantages of such drugs are (i) their activity against bacterial persisters and antibiotic-tolerant bacterial populations [13], characterized by a low metabolic activity [14,15]; (ii) their low tendency for the development of bacterial resistance [16]; and (iii) the fact that they do not need to cross the bacterial outer membrane (OM) of Gram-negative bacteria.

Indeed, this mode of action should limit the emergence of resistance through drug modifications by intracellular resistance-causing enzymes and efflux pumps expressed by multidrug-resistant (MDR) bacteria. Moreover, interaction with key membrane components, present in multiple copies in the bacterial membranes, can cause many antibacterial destructuring effects. The bacteria that are resistant to these effects have to perform biochemical modifications of multiple membrane components [17]. The corresponding metabolic modifications have a high energetic cost for the selected resistant bacteria, resulting in slow growth, rapid reversibility of the emerging resistance and high sensitivity to antibiotic drugs of other classes.

**Figure 2:**
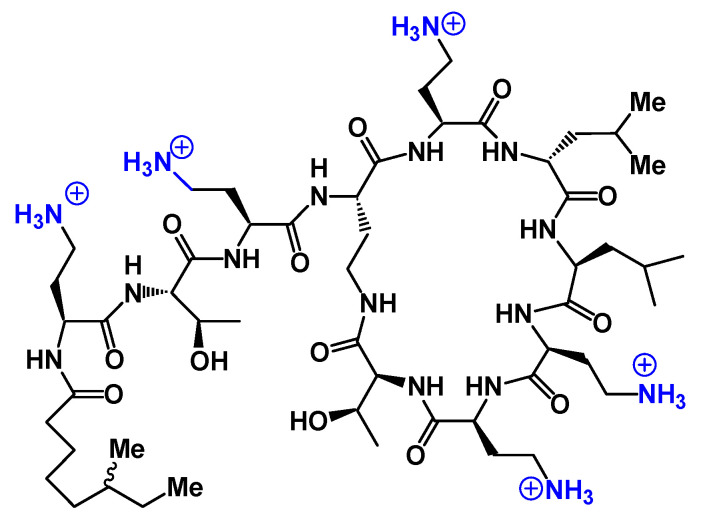
Structure of polymyxin E (COL), showing the five amine functions protonated at physiological pH.

In such an approach, the antibacterial potential of cationic amphiphilic compounds that target bacterial membranes is attractive from a drug development perspective. The repurposing of the old antibacterial cationic cyclopeptide polymyxin E, colistin (COL, Figure 2), acting on the bacterial outer membrane (OM), as a last-line antibiotic to treat multidrug-resistant (MDR) Gram-negative bacterial infections, especially those caused by antibiotic-resistant *Pseudomonas aeruginosa* strains, illustrate well the interesting nature of cationic amphiphilic antibiotic drugs [18]. Despite several studies on COL repurposing, many issues related to emerging bacterial resistances, toxicity and pharmacokinetics still need to be elucidated [18,19].

In the search for membrane-targeting antibiotics, we have focused our work on Gram-negative bacteria, such as *P. aeruginosa*, an opportunistic pathogen that causes a wide range of severe opportunistic infections in patients with serious underlying medical conditions, such as those with burns, surgical wounds or people with cystic fibrosis [20].

The interaction of cationic species with bacterial membrane components such as anionic phospholipids can produce membrane disruption and depolarization. The cell envelope of Gram-negative bacteria contains two membranes, the inner membrane (IM) and the OM, separated by the periplasm. The OM of Gram-negative bacteria has a unique architecture that acts as a potent permeability barrier against antibiotics. The OM is composed of lipopolysaccharide (LPS), phospholipids, outer membrane β-barrel proteins (OMPs) and lipoproteins. These components are synthesized in the cytoplasm or in the IM, and are then selectively transported to the OM by specific transport machines. Recent reviews on the transport and assembly systems of OM components have been published with the aim of developing inhibitors targeting these systems [21,22,23,24].

In the search for new antibacterial agents, we identified broad-spectrum antibacterial AAGs carrying two or three lipophilic groups and, for the first time, we revealed their effects on bacterial membranes [5,25]. There was a corresponding strong increase in the AG lipophilicity resulting in the bacterial target shift from rRNA to membranes and a significant improvement in activity against bacterial strains resistant to the parent AG drugs and to antibiotic drugs of other classes (penicillins, fluoroquinolones, macrolides, etc.). The identification of antibacterial AAGs acting on the bacterial membranes against AG-resistant bacteria and MDR bacteria offered a promising direction for the development of novel antibiotics. Most of the antibacterial AAGs identified were mainly active against susceptible and MDR Gram-positive bacteria and a few appeared to be active against susceptible and resistant Gram-positive and Gram-negative bacteria.

In the first part of this review article, we summarize the results that we obtained in the development of broad-spectrum antibacterial amphiphilic neamine (NEA) **6**, paromamine (PARA) **7** and 6-amino-6-deoxy-1-methylglucosamine (1-methyl neosamine, corresponding to ring II in NEO) derivatives (Figure 1), including some recent unpublished results, with a focus on the most active NEA derivatives. Structure–activity and structure–eukaryotic cytotoxicity relationships, as well as specificities and particularities in their mode of action and barriers that need to be crossed in their development as medicinal agents, are delineated. Since the integrity of the biophysical properties of bacterial membranes are required for maintaining their permeability functions, as well as the right environment for proteins embedded within, we explored the effects of AAGs on two major lipids, LPS and cardiolipin (CL), that are involved in one of the most critical biophysical characteristics of the OMs of Gram-negative bacteria, their asymmetry. LPS is located at the outer leaflet of the OM and CL is located mostly within the IM, and also within the OM [26]. Some selectivity results from the binding of AAGs to these lipids in bacterial membranes in comparison to mammalian ones, since bacterial membranes (i) are more negatively charged than eukaryotic membranes; (ii) contain a higher proportion of negative intrinsic curvature lipids, where proteins involved in the formation of the division plane are located [27,28]; and (iii) exhibit a dilational modulus of elasticity that is much lower than the one found in mammalian membranes [29].

At the end of the first part of this review article, other advances in the field of antibacterial AAGs, published since 2016 after the appearance of several review articles [7,8,30,31,32], are reviewed with an emphasis on AAGs made of an AG core conjugated to an adjuvant or an antibiotic drug of another class (antibiotic hybrids), as recently developed by Schweizer, Zhanel and coworkers.

Numerous other biological and biochemical effects of AAGs have been reported, and, in the second part of this article, we illustrate briefly some of these effects, i.e., their antifungal activity; their use as delivery vehicles of nucleic acids, of short peptide nucleic acids and of drugs; their ability to cleave DNA at abasic sites and their ability to inhibit the functioning of connexin hemichannels. Structure–activity relationships are finally discussed, in order to explain and improve their target selectivity.

## 2. Antibacterial Amphiphilic Aminoglycosides (Antibacterial AAGs)

The chemical strategies used for the preparation of new AGs and AAGs and their biological activities were recently reviewed [1]. The recent progress in AG conjugation for RNA targeting was also summarized in 2020 [33]. AAGs were synthesized by modification of the AG’s primary amine or hydroxyl functions. Lipophilic groups were conjugated to the AG’s core of NEO **1** (NEO), NEA **6**, PARO **2**, PARA **7**, KANA **3** and KANB **4**, TOB **5** and nebramine **8** (NEB) (Figure 1) using several strategies [3,4,5,6,7,8,30,31,32,33,34,35,36,37,38]. The amine functions of the selected AG were converted to alkyl- or aryl-amide(s) or to carbamates, leading to a decrease in the number of positive charges present at physiological pH. In another approach, the hydroxyl groups were converted to ether and thioether groups. Such modification(s) of the amine or hydroxyl function(s) of the AG cores should produce AAGs that are not a substrate of AG-deactivating enzymes (AG nucleotidyltransferases (ANTs), AG phosphotransferases (APHs) and AG acetyltransferases (AACs) [2], and therefore produces AAGs which are active against bacteria expressing resistance-causing enzyme(s).

The AAGs identified to be most active against Gram-positive and Gram-negative bacteria are mainly dialkyl and/or trialkyl derivatives of NEA **6** (Figure 1), [5,34,35], of 1-methyl neosamine [36] and of NEB **8**, which were identified more recently and are briefly described [37,38]. In this part of the review, we summarize the main results obtained in the development of broad-spectrum antibacterial amphiphilic NEA **6**, PARA **7** and 1-methyl neosamine derivatives. Some physicochemical properties of amphiphilic NEA derivatives and a method developed for their dosage are also reported (unpublished results).

### 2.1. Broad-Spectrum Antibacterial AAGs: Antibacterial NEA, PARA and 6-Amino-6-Deoxy-1-Methylglucosamine Derivatives, Structure–Activity and Structure–Cytotoxicity Relationships, Modes of Action

#### 2.1.1. Synthesis of NEA and PARA Derivatives

Our first works in the field of AGs were developed in the search for anti-HIV agents targeting viral RNA [39]. The AG core of NEA **6**, carrying four amine and four hydroxyl functions, has been selected for modification in order to reduce the number of cationic groups present in the synthesized AAGs and, as a consequence, to limit the unspecific binding of the resulting species to biological polyanionic components.

Methods of protection and deprotection of amine and hydroxyl functions of NEA **6**, obtained by methanolysis of NEO **1**, were developed. From *N*-tetratritylated NEA **9**, the 3′,6-*O*-di-*para*-methoxybenzyl (diPMB) and 3′,4′,6-*O*-triPMB derivatives **10** and **11** (Figure 1) were prepared for selective *O*-alkylation and deprotection under acidic conditions to give 4′-,5-*O*-monoalkyl and 4′,5-*O*-dialkyl derivatives [39,40]. The *O*-alkylation of *N*-tetratritylated NEA **9** in DMF using sodium hydride (NaH) as a base allowed the preparation, in one step, of 3′,6-*O*-dialkyl and 3′,4′,6-*O*-trialkyl NEA derivatives [5]. Using phase transfer conditions, it was possible to prepare, in high yields, the 6-PMB tetratrityl NEA derivative **12**, which was alkylated to selectively produce 3′,4′-*O*-dialkyl derivatives [36,41]. Methanolysis of PARO **2** gave PARA **7,** which was tetratritylated, leading to **13**, which was used to prepare 3′,6-*O*-dialkyl and 3′,4′,6-*O*-trialkyl PARA derivatives [34]. Major increases in the selectivity and/or reactivity of AAGs in *O*-mono- and *O*,*O*′-di-alkylation were observed under phase-transfer conditions in the presence of tetrabutylammonium fluoride (TBAF) or iodide (TBAI) [41] in comparison to alkylation in DMF solution using NaH [5]. The use of TBAI under phase transfer conditions allowed the rapid and efficient preparation of 3′,6-dialkyl and 3′,6-diarylalkyl NEA derivatives, avoiding the *O*-trialkylation observed with NaH in DMF [5,33,34].

#### 2.1.2. Structure–Activity Relationships

##### First Identified Broad-Spectrum Antibacterial Amphiphilic NEA Derivatives

**Figure 3:**
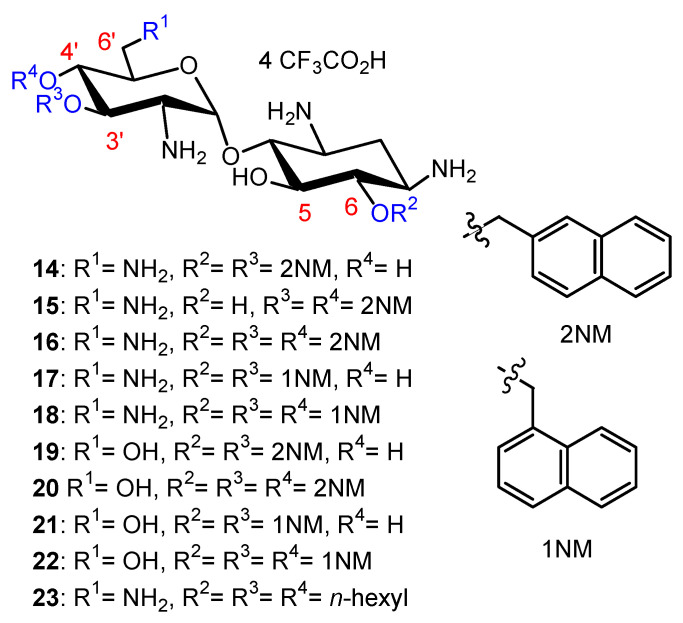
Structures of the first identified broad-spectrum antibacterial amphiphilic neamine (NEA) (**6**) and paromamine (PARA) (**7**) derivatives [5,34].

In the search for new antibacterial agents, first, we introduced onto the hydroxyl functions of NEA lipophilic benzyl, 2-naphthylmethylene (2NM) (Figure 3), 2-pyridinylmethylene and 2-quinolinylmethylene groups from the corresponding halides [5]. The resulting 3′,6-di-; 3′,4′-di- and 3′,4′,6-triarylalkyl NEA derivatives were isolated as tetratrifluoroacetate salts, after a final deprotection step in TFA, and were evaluated for their antibacterial activity against susceptible and resistant Gram-positive *Staphylococcus aureus* (for instance, methicillin-resistant *S. aureus* (MRSA) and vancomycin-resistant *S. aureus* (VRSA)) and Gram-negative bacteria (*Acinetobacter baumannii*, *Escherichia coli, Klebsiella pneumonia, P. aeruginosa*, etc.).

The 3′,6-di-(**14**); 3′,4′-di-(**15**) and 3′,4′,6-tri-(**16**) 2-naphthylmethylene (2NM) derivatives (Figure 3) showed good activity against susceptible and resistant *S. aureus* strains expressing resistance pumps (NorA or MsrA), against AG-inactivating enzymes and against MRSA and VRSA strains, which are resistant to methicillin and vancomycin, respectively (minimum inhibitory concentrations (MICs) 4–16 µg/mL), whereas the 3′-, 4′-, 5′-, 6-mono-2NM derivatives were inactive (MICs > 128 µg/mL). The tri2NM derivative **16** showed better antibacterial activity against the selected Gram-positive strains (MICs 2–4 µg/mL). It also exhibited good activity (MICs 4–16 µg/mL) against sensitive and resistant Gram-negative bacteria, both on Enterobacteriaceae and non-Enterobacteriacea, including *P. aeruginosa,* expressing efflux pumps or AG-deactivating enzymes, or rRNA methylases [2] (*Citrobacter amalonaticus* arm 06AB0010, *E. coli* 06AB003arm, *Enterobacter aerogenes* 06AB008 arm). Derivatives **14** and **16** revealed weak and aspecific binding to a model bacterial 16S rRNA as compared to NEO **1**. Derivative **16** also showed a low ability to decrease ^3^H leucine incorporation into proteins in *P. aeruginosa*, suggesting that **16** acts against Gram-negative bacteria with a mechanism different from inhibition of protein synthesis, probably by membrane destabilization [5]. For the first time, AAGs (**14** and **16**) were shown to target the membranes of *P. aeruginosa* with induction of depolarization [25].

##### Comparison of the Antibacterial Activities of NEA and PARA Derivatives

In the PARA core, the 6′-amino group of NEA is replaced by a hydroxyl group and, as a consequence, the corresponding AAGs bear, at physiological pH, one less positive charge in comparison to their NEA homologs [34]. The comparison of the antibacterial activities of the previously prepared 2NM derivatives (**14** and **16**) with those of 1-naphthylmethylene (1NM, **17** and **18**) NEA derivatives and of the corresponding PARA derivatives **19**–**22** (Figure 3), revealed the better good broad-spectrum activity of the 3′,4′,6-triNM NEA derivatives, with the MIC mainly decreased by four times. Therefore, the NEA core was selected in the search for more active compounds than **14**–**18** [34]. The presence of an amino group at position 6′ that is protonated at physiological pH of the aminoglycoside increases the antibacterial effect, without being essential to the antibacterial effect.

**Figure 4:**
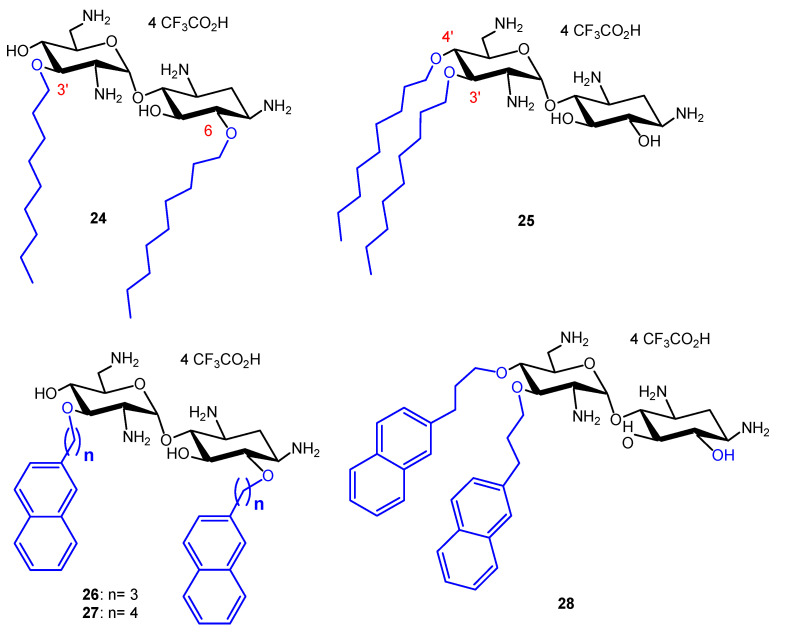
Structures of the identified broad-spectrum antibacterial amphiphilic dialkyl (**24** and **25**) and dialkylnaphthyl (**26**–**28**) NEA derivatives [34].

In order to compare the antibacterial activity of 3′,6-dialkyl and 3′,4′,6-trialkyl NEA derivatives and the effect of the alkyl chain lipophilicity on their activity, NEA derivatives carrying two or three linear alkyl groups, butyl (Bu), hexyl (Hx), nonyl (Nn) and octadecyl (Ocd) groups, as well as arylalkyl groups, benzyl and 2-naphthylalkyl with alkyl, methyl, *n*-propyl, *n*-butyl and *n*-hexyl (2NM, 2NP, 2NB and 2NH, respectively), were synthesized and evaluated [34]. The 3′- and 6-mono-Ocd derivatives were also prepared and found to be inactive against Gram-positive and Gram-negative bacteria. The 3′,4′,6-tri2NM (**16**), -triHx (**23**) (Figure 3) and the 3′,6-diNn (**24**), -di2NP (**26**) and -di2NB (**27**) NEA derivatives (Figure 4) showed good activity against susceptible and resistant Gram-positive and Gram-negative bacteria, with the 3′,6-dinonyl derivative **24** being the most active, and the di2NP **26** and di2NB **27** also being the most active against Gram-positive bacteria. The main result of this study was the delineation of windows of critical lipophilicity values for the active 3′,6-dialkyl and 3′,4′,6-trialkylNEA derivatives from the plot of 1/MIC versus clogP (calculated octanol/water partition coefficient; see the paragraph below on the fine-tuning of the structure–activity relationships). NEA AAGs **24**, **26** and **27** were selected for further study as the most active and the less cytotoxic derivatives at 10 μM to murine J774 macrophages, in comparison to the trihexyl derivative **23** that has shown slightly lower broad-spectrum activity [34]. These three AAGs were shown to be more active against COL-resistant *P. aeruginosa* (PA272, PA307, PA343 and PA2938; MIC = 2–8 µg/mL) than the 3,’4′,6-tri2NM NEA derivative **16** (MIC = 4–32 µg/mL) [42].

AG antibiotics are effective against biofilms and recently approaches and possible mechanisms for the application of AGs to treat biofilm-associated infections were briefly reviewed [43].

The 3′,6-dinonyl NEA derivative **24** was shown to be bactericidal against *P. aeruginosa* at its MIC and to inhibit the *P. aeruginosa* biofilm formation at a two-fold MIC [42].

##### Comparison of the Antibacterial Activities of 3′,6- and 3′,4′-Dialkyl NEA Derivatives

In order to investigate the role of the attachment position of the second alkyl chain in antibacterial activities and cytotoxicities, the 3′,4′-diNn and -di2NP analogues **25** and **28** of the corresponding 3′,6-dialkyl derivatives **24** and **26** (Figure 4), were synthesized from 6-(*para*-methoxy)benzyl-*N*-tetratrityl NEA **12**, obtained selectively under phase transfer conditions of alkylation [36,41]. Against susceptible and resistant Gram-positive bacteria, the 3′,6-di2NP and 3′,4′-di2NP derivatives **26** and **28** showed similar good activity. Against Gram-negative bacteria, the activity of **28** was better. The 3′,6-dinonyl derivative **24** appeared to be more active than its 3′,4-dinonyl isomer **25** and was found to be the most active synthesized NEA derivative against susceptible and resistant Gram-positive and Gram-negative bacteria [36].

##### 6-Amino-6-Deoxy-1-Methylglucosamine (1-Methyl Neosamine) Derivatives, Analogues of 3′,4′-Dialkyl NEA Derivatives

**Figure 5:**
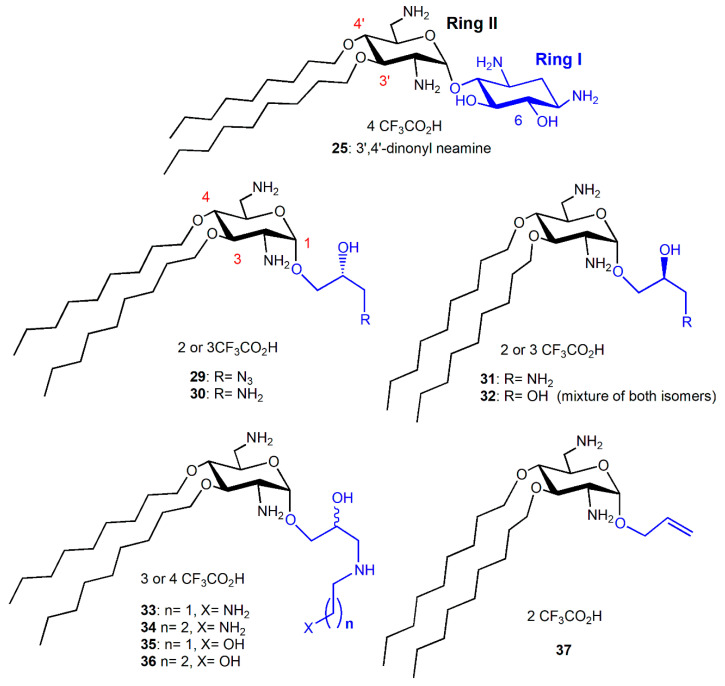
Structures of the broad-spectrum antibacterial 3′,4′-dinonyl NEA derivative and of the corresponding antibacterial analogues synthesized in the 1-methyl neosamine series [36].

In order to evaluate the importance, in the antibacterial activity of 3′,4′-dinonyl derivative **25**, of the integrity of the 2-deoxystreptamine ring I (Figure 5), amphiphilic 3,4-dialkyl derivatives **29**–**36** of 6-amino-6-deoxyglucosamine, named neosamine, corresponding to NEA ring II, were synthesized from the 1-allyl intermediate derivative **37**, prepared from *N*-acetylglucosamine (in 8 or 9 steps) (Figure 5) [36]. The most hydrophilic derivatives **30**–**36** mainly showed similar good activity against susceptible and resistant Gram-positive and Gram-negative bacteria (MIC = 1–2 and 4–8 µg/mL, respectively), slightly better than the corresponding activity of the 3,4′-dinonyl NEA derivative **25**. The azido derivative **29** revealed an activity against Gram-positive bacteria similar to **30**–**36** but was mainly inactive against Gram-negative bacteria (MIC = 32–>128 µg/mL). The major difference observed in comparison to the 3′,6- and 3′,4′-dinonyl NEA derivatives, **24** and **25**, was a better activity against the resistant *Acinetobacter lwoffi* strain Al88-483 of **30**–**32** and **34**–**36** (MIC = 4–8 µg/mL), the only selected bacteria against which **24** revealed an MIC value higher than 2–4 µg/mL (32 µg/mL). These results showed that the integrity of ring I is not necessary to have good broad-spectrum antibacterial activity. They suggest that, at least, one amine function, protonated at physiological pH, should be present in the flexible chain replacing ring I, since the azido derivative **29** is mainly inactive and the hydroxyl derivative **32** is less active than **31** and **33**–**36** against susceptible and resistant Gram-negative bacteria, especially against *P. aeruginosa* strains (MICs = 64 and 4–8 µg/mL, respectively).

##### Fine-Tuning of the Structure–Activity Relationships (SAR)

In the previous studies, the 3′,6-dinonyl NEA derivative **24** has been found to be the most active AAG against susceptible and resistant Gram-negative bacteria with the exception of resistant *A. lwoffi* (Al88–483). In the search for the best antibacterial amphiphilic NEA derivatives to develop further, different 3′,6- and 3′,4′-dialkyl NEAs having lipophilicity values close to that of **24** were synthesized and studied [35].

**Figure 6:**
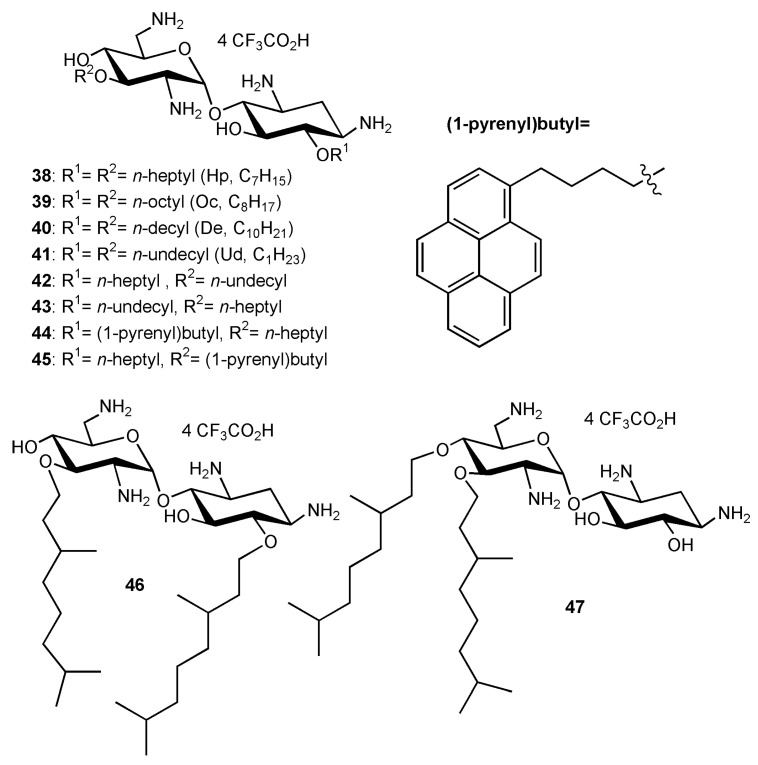
Structures of antibacterial homodialkyl (**38**–**41**, **46**, **47**) and heterodialkyl (**42**–**45**) NEA derivatives synthesized [35].

3′,6-homodialkyl NEA derivatives carrying *n*-heptyl (Hp, C7); *n*-octyl (Oc, C8); *n*-decyl (De, C10) and *n*-undecyl (Ud, C11) groups, **38**–**41**, respectively, were prepared (Figure 6). Two 3′,6-heterodialkyl derivatives, 3′-*n*-heptyl-6′*n*-undecyl (Ud, C11) **42** and 3′-*n*-undecyl -6′-*n*-heptyl (Ud, C11) **43**, and two dialkyl derivatives bearing the same branched chain, 3′,6-di(3,7-(dimethyl)octyl (diDiMOc) NEA **46** and its 3′,4′-isomer **47**, were also synthesized (Figure 6). Two 3′,6-heterodialkyl NEA derivatives, **42** and **43**, having lipophilicities close to that of **24** (3′- or 6-heptyl and 6- or 3′-undecyl) were synthesized in order to evaluate the effects of the presence of dissymmetric alkyl chains on the antibacterial effects and the eukaryotic cytotoxicity. Two fluorescent 3′,6-dialkyl NEA derivatives, **44** and **45**, carrying one *n*-heptyl group and one (1-pyrenyl)butyl group, and also having lipophilicities close to those of **24**, were also prepared as probes for mechanistic studies (Figure 6).

**Figure 7:**
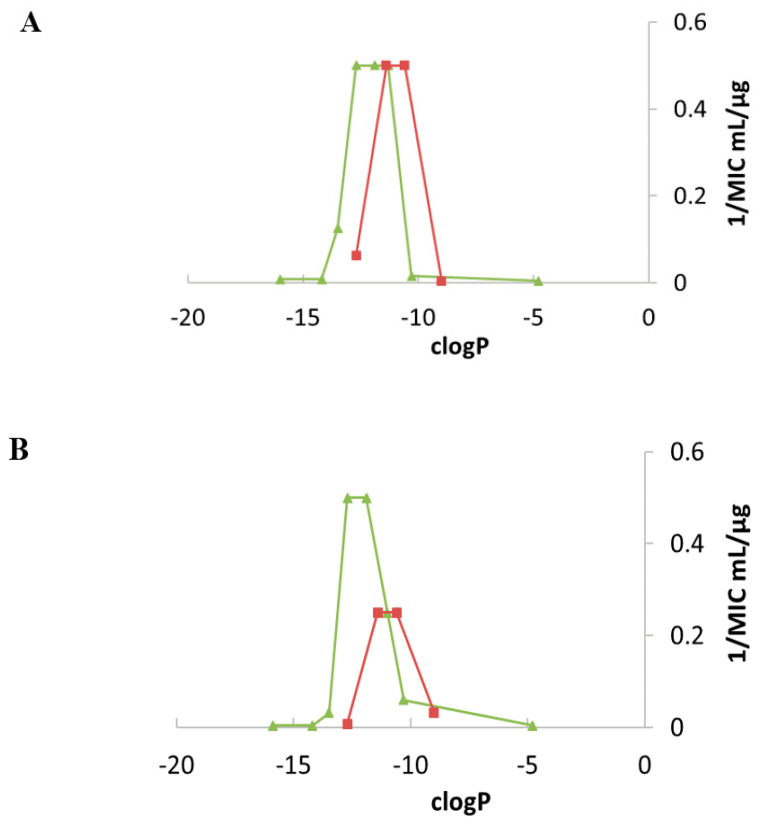
Values of 1/(MIC (mL/µg) as a function of clogP values for 3′,6-dinaphthylalkyl NEAs (di2NM **14**, di2NP **26**, di2NB **27** and di2-naphthylhexyl) and 3′,6-dialkyl NEAs (diC4, diC6, diC7 **38**, diC8 **39**, diC9 **24**, diC10 **40**, diC11 **41** and diC18). (**A**) Against MRSA; (**B**) against susceptible *P. aeruginosa* ATCC 27853. Naphthylalkyl derivatives: red squares; alkyl derivatives: green triangles [35].

Two windows of lipophilicities have been related to the antibacterial activities of the 3′,6-dialkyl and -dialkylaryl derivatives and to the 3′,4′,6-trialkyl and –trialkylaryl derivatives, respectively, through the plot of 1/MIC versus clogP (calculated octanol/water partition coefficient) [34]. The fine-tuning of the delineated critical window of lipophilicity related to the antibacterial activity of the 3′,6-dialkyl and -dialkylaryl derivatives was possible with the new 3′,6-dialkyl derivatives synthesized [35]. It revealed differences between the series of dinaphthylalkyl NEAs and the series of homodialkyl NEAs (Table 1, Figure 7) [35]. Against *P. aeruginosa*, the dinaphthylpropyl derivative **26** is slightly less active than the alkyl derivatives **24** and **39**, despite close lipophilicities. Against Gram-positive bacteria, the activity is similar (MICs 1–2 µg/mL). The dinaphthylalkyl derivative **26** is less cytotoxic (Table 2) than the dialkyl derivatives **24** and **39** at 30 µM. Fridman and coworkers reported that an increased degree of unsaturation in the lipophilic chain of antifungal AAGs derived from TOB **5** significantly reduced the immediate toxicity to mammalian cells in comparison to saturated derivatives [44].

In [35], four new broad-spectrum antibacterial derivatives of similar lipophilicities were identified, two 3′,6-dialkyl derivatives carrying two linear octyl and decyl chains **39** and **40**, and, 3′,6- and 3′,4′-dialkyl derivatives bearing the same branched 3,7-(dimethyl)octyl (DiMOc) chains **46** and **47**. In comparison to **24** and **25**, this study revealed that the grafting of branched alkyl chains of similar lipophilicities (NEAs **46** and **47**) preserves or increases the antibacterial activity and increases the eukaryotic cytotoxicity. 3′,6-heterodialkyl NEAs **42**–**43**, of lipophilicities close to those of **24** and **25**, showed good broad-spectrum antibacterial activity, and it was shown that the dissymmetry of their chains increases the cytotoxicity. The fluorescent pyrenyl derivatives **44** and **45**, having good antibacterial activity, are under study for related mechanistic studies (unpublished results).

The previously identified 3′,6-dinonyl NEA **24** and the 3′,6-dioctyl NEA **39**, identified in this study, appeared to be the most active amphiphilic NEAs against susceptible and resistant Gram-positive and Gram-negative bacteria (for example, Table 1) and the least cytotoxic broad spectrum antibacterial 3′,6-dialkyl derivatives (without aryl groups) to mammalian cells (Table 2). These derivatives and the 3′,6-diarylalkyl NEA derivative **26**, which showed a better activity against Gram-positive than against Gram-negative bacteria and is the less active cytotoxic derivative at 30 µM (Table 1 and Table 2), merit further interest in an antibacterial development.

##### Emergence of Resistance to Amphiphilic NEA Derivatives: MIC Changes against *P. aeruginosa* upon a Long Exposures to AAGs

We studied, in comparison to ciprofloxacin (CIP), the MIC changes against susceptible *P. aeruginosa* ATCC 27,853 upon several days’ exposure to a half-MIC of 3′,6-di2NP NEA **26** and its 3′,4′-isomer **28** [36], and to the 3′,6-diNn NEA derivative **24** [35].

Exposure of *P. aeruginosa* to subinhibitory concentrations of CIP or di2NP NEAs caused a decrease in susceptibility that appeared later for the di2NP derivatives. The exposure of susceptible *P. aeruginosa* to a half-MIC of **24** at different times, over more than one month, also demonstrated the expected high difficulty of resistance emergence to AAGs, with a much weaker and slower increase of MIC in comparison to CIP. Under these conditions, the MIC increased slightly from 1 to 4 µg/mL at day 15, 30 and 38 as compared to the MIC values of CIP, which increased faster, from 0.5 to 16 µg/mL at days 6, 21 and 24 [35].

##### Solubility of AAGs at High Concentration in Aqueous Solutions and Dosage of 24 for Studies In Vivo (Unpublished Results)

The synthesized AAGs were isolated and evaluated mainly as tetratrifluoroacetates of high Mw (**24**: 1031 g/mol; **26**: 1115 g/mol) isolated after lyophilization as white foams. They are hygroscopic and highly soluble in water, at more than 50 mM for **24** and **26**, for example. The solubility S decreases strongly in the presence of glucose (S < 5 mM), of 0.9% aqueous NaCl (S < 3 mM) and of 1× phosphate-buffered saline (PBS) aqueous solution (S < 1 mM). This behavior corresponds to a difficulty in the in vivo antibacterial evaluation of **24** and **26**, especially through intravenous injections at high concentration.

The dinonyl derivative **24** does not incorporate a chromophore absorbing in the near and medium UV useful for dosage. Therefore, a spectrofluorimetric method of dosage was optimized by derivatization of **24** with fluorescamine from a method described to quantify the concentration of AGs such as NEO **1** [45,46], KAN and TOB **5** [46,47]. In the reported method, acetone has been used as a solvent of fluorescamine. It was replaced by less volatile acetonitrile, resulting in an increased fluorescence intensity that was found to be linearly correlated to the concentration of the dinonyl derivative **24**, in the range of 1 to 30 µM.

#### 2.1.3. Targets and Modes of Action against Gram-Negative Bacteria

In the search for new antibacterials and for the fine-tuning of the most promising AAGs, the understanding of the molecular mechanisms involved in the antibacterial activity is critical. The OM of Gram-negative bacteria has a unique architecture that acts as a potent permeability barrier against antibiotics. Since the biophysical characteristics of bacterial membranes, and especially OM asymmetry, are required to maintain their permeability functions as well as the right environment for proteins embedded within, we focused our studies on the effect of AAGs on the two lipids involved in membrane asymmetry, LPS and cardiolipin.

##### Anionic LPS

**Figure 8:**
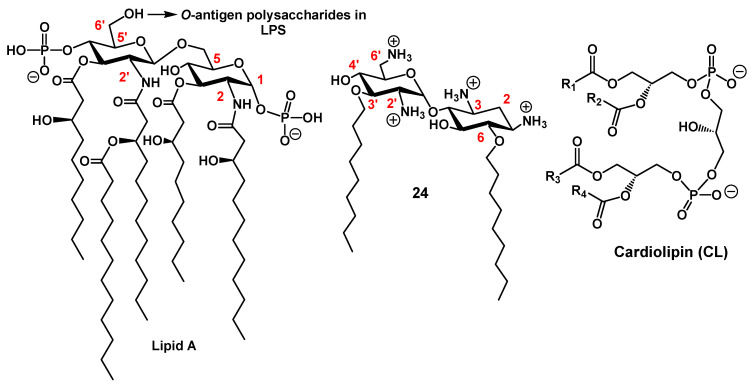
Comparison of the structures of lipid A and cardiolipin (CL) to the structure of the antibacterial 3′,6-dinonyl NEA derivative **24**.

LPS is an essential component of the OM of many Gram-negative bacteria. LPS is a complex macromolecule, comprising the endotoxic lipid A (Figure 8), covalently linked to a polyphosphorylated oligosaccharide core carrying an *O*-polysaccharide chain (also referred as *O*-antigen) [48]. The lipid A core serves not only as a hydrophobic anchor of LPS in order to form the bilayer structure, but also as an activator of the innate immune system [48]. This core is made of the diphosphorylated 1,6-*N*-acetylglucosamine dimer *O*- and *N*-acylated with 4–7 fatty acids (Figure 8). 

Lipid A structural variations (mainly in acylation patterns) depend upon bacterial species [49]. As an example, in *P. aeruginosa*, LPS (LpxA) incorporates C10 lipophilic chains grafted on UDP-*N*-acetylglucosamine, whereas C14 and C12 chains are incorporated in *E. coli* and *Neisseria meningitidis*, respectively [50].

In the continuous search for membrane-targeting antibiotics, compounds that interfere in LPS biogenesis or regulatory pathways offer opportunities for development as antibiotics that may be useful against pathogenic bacteria [22,23,51,52].

The electrostatic attraction between the amphiphilic NEA derivatives and the negatively charged lipid or LPS headgroups (i.e., core oligosaccharide and Lipid A phosphates in LPS) is likely the dominant driving force of the interaction [53,54]. However, other parameters governing the interactions between LPS and amphiphilic NEA derivatives, including (i) the area per lipid, (ii) the ordering and the hydration behavior, (iii) the spatial conformation and (iv) the molecular shape of the lipid A moiety, also play a critical role and are discussed hereunder.

First, the **area per lipid of the lipid A molecule** is critical for characterizing the LPS/lipid A packing. The acyl chains in lipid A occupy a smaller volume than in most of the phospholipids and could be associated with a better packing [55]. Especially, in the *P. aeruginosa* membrane, the tail volume of the penta-acyl lipid A is smaller as compared to that found in other bacteria having hexa- or hepta-acyl chains in their membranes, such as *Salmonella minnesota* [55,56,57].

Amphiphilic NEA derivatives were inserted into LPS monolayer, as indicated after spreading them in the subphase and measuring the compression isotherms of rough mutant. A shift to molecular areas higher than those for the pure buffer subphase was observed [42]. This results in a higher packing. Addition of 3′,6-dinonyl NEA **24** in the presence of Ca^2+^ gave rise to an effect on the molecular area that was weaker than the effect obtained in the absence of Ca^2+^, suggesting that CaCl_2_ reduced the insertion of the 3′,6-dinonyl NEA derivative [42].

As mentioned, and demonstrated by displacement assays, it is not only electrostatic interactions that are involved in the binding of amphiphilic NEA derivatives to LPS—the length and branching of acyl chains in LPS are also critical. Different 3′,6-dialkyl NEA derivatives were found to bind to LPS with a clear ranking, depending upon the length of the linear alkyl chain (3′,6-diheptyl NEA **38** < 3′,6-dioctyl NEA **39** < 3′,6-dinonyl NEA **24** < 3′,6-didecyl NEA **40** < 3′,6-undedecyl NEA **41**). The presence of a branched alkyl chain also plays a critical role (3′,6-dinonyl NEA **24** < 3′,6-di(dimethyloctyl) NEA **46** (Swain et al., unpublished results). 

Second, closely interconnected with LPS/lipid A packing, the **order and lipid phase** might also be key clues for the interaction between LPS/lipid A and amphiphilic aminoglycoside antibiotics. The order parameter of lipid A from *P. aeruginosa* can be determined using Fourier-transform infrared (FT-IR) spectroscopy [58]. The antibacterial amphiphilic NEA derivatives make the LPS film state more liquid-like, as suggested by the decrease in the excitation generalized polarization (GPex) of a fluorescent laurdan probe (6-dodecanoyl-2-dimethylamine-naphthalene), which is sensitive to lipid hydration, inserted into LPS micelles [59,60]. This indicates an increasing liquid-like state in the lipid A region of LPS. The effect was dependent upon the presence of aryl groups (3′,6-di2NP **26** < 3′,6-diNB **27** < 3′,4′,6-tri2NM **16**). The effect induced by 3′,6-dinonyl NEA **24** was not significantly different from that observed with 3′,6-diNP **26**, of similar lipophilicity. In contrast, COL, NEA and the 3′,6-di2NM derivative **14** had no influence on the packing of lipid A in micelles, as indicated by unchanged GPex values compared to the value for the control. The appearance of fluidic material extends previous data related to the formation of a supramolecular network between multi-cationic and multi-anionic substances [61,62]. The liquid-like state in the lipid A region of LPS was likely more predominant than observed with glycerophospholipid. Divalent calcium ions could form salt bridges due to the close proximity of lipid A phosphate groups [63]. This binding is tight enough that the charge density in the solvent can approach zero [55]. All kinks in the water density lines, which indicate the enhanced water penetration, are located around z = 8 nm in the vicinity of the peaks of both lipid A phosphates and calcium ions [55]. The presence of divalent cations explains why the water molecules are able to penetrate deeper into the lipid A leaflet than the phospholipid leaflet.

The **conformation of lipid A** could be the third critical parameter for designing new potential antibiotics. The spatial conformation of lipid A is an indicator of lipid A bioactivity [58,64,65]. The backbone inclination angle of asymmetric penta-acyl lipid A influences the position and orientation of the phosphate groups in LPS. Other inclination angles also provide information to further determine the lipid A conformation, such as the angles between the membrane surface and the vectors from 4′-carbon to 4′-phosphorus, 1-carbon to 1-phosphorus and 4′-carbon to 1-phosphorus atoms [55]. This might help us to design new positions for substitution or new branching.

Lastly, the **molecular shape** of LPS/lipid A could play a critical role in its interaction with amphiphilic NEA derivatives. The penta-acyl LPS has a multilamellar structure due to its cylindrical molecular shape. To be biologically appropriate (for interaction with Toll-like receptors, for example), this structure has to be converted into a physiologically active conformation [58]. The transition from a unilamellar into a cubic inverted structure suggests that a subsequent intercalation into the hydrophobic moiety takes place, resulting in a change of the molecular shape of the LPS/lipid A molecules with a more conical conformation [66].

Assuming the volume of the hydrophobic part increases from the linear dialkyl (3′,6-dinonyl NEA **24**) to the dinaphthylalkyl (3′,6-di2NM NEA **14**, 3′,6-di2NP NEA **26**, 3′,6-di2NB NEA **27**) and the bulky 3′,4′,6-tri2NM NEA **16** moieties, the maximal binding would be inversely proportional to the volume of the hydrophobic moiety. The 3′,6-dinonyl NEA **24** shows a molecular shape of an inverted cone, with a large hydrophilic part and a small hydrophobic one. This could allow a close interaction between this derivative and the lipid A moiety of the LPS unit, characterized by a conical complementary shape (with a small hydrophilic part and a large hydrophobic part). Very interestingly, LPS acylation can be modified through the activity of the palmitoyl lipid A transferase PagP, leading to new modulation of LPS activity [67].

In summary, the small area per lipid and large order parameter of lipid A indicate a leaflet with low fluidity and highly organized molecules. Calcium ions form salt bridges with phosphates in lipid A and stabilize the corresponding structure. The overall hydrated lipid A leaflet displays solvation differences due to the tilted lipid A backbone. This inclination causes the 1-phosphate in lipid A to project toward the outer aqueous environment and the 4′-phosphate to become buried between the acyl chains of lipid A [55]. Studies have highlighted the formation of a fluidic cross-linked supramolecular network between LPS and amphiphilic NEA derivatives, in parallel with the length and the logP values of the corresponding derivatives. Both hydrophobic and electrostatic interactions are required.

The molecular shape of the 3′,6-dialkyl NEA derivatives induced by the nature of the grafted hydrophobic moieties (naphthylalkyl instead of alkyl) and the flexibility of the hydrophobic moiety are critical for their fluidifying effect and their ability to displace cations bridging LPS. These parameters could be exploited for the development of new amphiphilic NEA derivatives [42].

How these characteristics in the interactions between LPS and amphiphilic NEA derivatives result in antibacterial activity is still unclear. Activity was associated with the self-promoted insertion of the molecule within the lipid A region of the OM outer leaflet in *P. aeruginosa*, leading to an increase in OM permeability. The fluorescent probe 1-*N*-phenylnapthylamine (NPN) was used to probe the permeability of the OM, since NPN is a small molecule that cannot effectively cross the OM. It is weakly fluorescent in aqueous solution but fluoresces strongly when it binds to phospholipids [68].

An ascending time dependence of the Ca^2+^ effect in NPN uptake experiments was observed from 3′,6-di2NM NEA **14** to 3′,6-di2NP NEA **26** and 3′,6-diNB NEA **27** [42]. For linear dialkyl NEA derivatives, no clear effect corresponding to the increase of side chain lengths was observed. Regarding the effect of branched alkyl groups present in the 3′,6-di(dimethyloctyl) derivative **46**, the dose required to obtain 50% of the maximal effect is lower for the branched derivative as compared to the unbranched analogues, and even the maximal effect was equal for both compounds (Swain et al., unpublished results).

##### Cardiolipin (CL)

CL (Figure 8) is a tetra-acylated diphosphatidylglycerol derivative found in plants and animals, but also in bacteria. This structurally unusual phospholipid carries two negative charges due to its dimeric structure, consisting of two phosphatidyl residues connected by a glycerol bridge and four associated fatty acyl chains (made of various alkyl chains R^1^, R^2^, R^3^ and R^4^) [69]. Due to variability in fatty acyl chains (length, degree of unsaturation, etc.), CL is characterized by numerous molecular species, leading to a high potential for adaptability or responses to stress. CL is known for its sensitivity to stressors, such as the addition of organic solvents or high salt content or the presence of quaternary ammonium compounds [70,71]. Changes in the molecular species suggest CL restructuring. Indeed, CL is a critical phospholipid in maintaining the function and morphology of bacteria. CL was one of the main components of the IM, along with phosphatidylethanolamine (PE) and phosphatidylglycerol (PG) [72,73]. Very interestingly, CL is also located in the OM of most Gram-negative bacteria [26,74].

Like LPS, CL is characterized by its own membrane parameters, including (i) molecular area, (ii) degree of unsaturation and length of acyl chains and (iii) the ability to induce negative curvature and form microdomains, parameters which are critical in the biophysical properties of membranes in which CL is inserted.

With the aim of characterizing the effect of the interactions between amphiphilic NEA derivatives with CL on the molecular area, we determined the compression isotherms corresponding to the lipid monolayers for CL spread on a subphase containing 3′,6-dinonyl NEA **24** (as compared to those of 1-palmitoyl-2-oleoyl-sn-glycero-3-phosphatidylethanolamine (POPE) and 1-palmitoyl-2-oleoyl-*sn*-glycero-3-phospho-(1′-*rac*-glycerol) (POPG)). The isotherms were shifted to higher molecular areas than the pure buffer subphase, although to a much lower extent for POPE, compared with POPG and CL. At 30 mNewtons/m, a value close to the estimated surface pressure of biological membranes in vivo, the mean molecular area for POPE was not significantly modified by the presence of 3′,6-dinonyl NEA **24** in the subphase. With POPG and CL, the mean molecular area increased by 1.8 and 1.6, respectively. This discrepancy indicates a much higher adsorption of **24** into POPG or CL than into POPE. The shift of the mean molecular area was not significantly different between POPG and CL.

Furthermore, **order and interdigitation** could be affected by AAGs. Bacterial CL structure is characterized by a high degree of symmetry and unsaturation [71,75,76], as well as by long fatty acyl chains. Both the degree of unsaturation and the length of acyl chains can modulate order and crosstalk between leaflets [77,78,79]. CL might be a reservoir for unsaturated fatty acid chains, as suggested by the content of unsaturated fatty acid in *cls*/*cls2*-mutant cells, which is approximatively 40% lower than in wild-type cells [71]. The relative concentrations of C16 and C18 for the barotolerant *Pseudomonas sp.* BT1 were approximately 60% and 40%, respectively [72]. PE and CL have a phase transition well above that of PG when the fatty acid content is identical for both lipids. In general, lipids with long fatty acyl chains undergo phase transition at higher temperature than lipids with short chains [80]. Long chains explain why CL stimulates changes in the physical properties of the membrane and why it decreases the lateral interaction within the monolayer leaflet, which favors the creation of membrane folds [81].

Another striking characteristic of CL is the comparatively small cross-section of its headgroup relative to the cross-section of its four large tail groups. This discrepancy results in a molecular shape with a large **intrinsic negative curvature** [82,83,84,85] and a non-bilayer anionic phospholipid also described previously for PE [86] that facilitates the insertion of membrane proteins. The cross-sectional size difference further explains the location of CL-enriched regions at the pole and/or the division septum [87], which can in turn be related to the polar localization of many proteins, including those involved in cell division and osmosensing [88]. The intrinsic negative curvature of CL can result in the formation of microdomains (clusters).

The presence and the role of these microdomains can be affected by membrane-acting antibiotics. Using membrane models mimicking *P. aeruginosa* plasma membrane composition (POPE:POPG:CL), we demonstrated the ability of 3′,6-dinonyl NEA **24** to induce in a CL dependent manner, to increase membrane permeability through reduced hydration and decreased ability of the membrane to mix and fuse, as shown by monitoring calcein release, GPex of laurdan and fluorescence dequenching of octadecyl rhodamine B, respectively [89]. After incubation with 3′,6-dinonyl NEA **24**, CL was co-localized with PE in small clusters, sections with increased membrane curvature, and fusion points in the joined giant unilamellar vesicles (GUV) population. Moreover, a clear overall lipid reorganization was observed [89].

CL-interacting proteins and functions regulated by CL are affected by the amphiphilic AG, as we demonstrated an inhibition of the respiratory chain and changes in bacterial shape. The latter effect was characterized by the loss of the bacterial rod shape through a decrease in length and an increase in curvature. It resulted from the effect on MreB, a CL-dependent cytoskeleton protein, as well as a direct effect of 3′,6-dinonyl NEA **24** on CL [90]. In *E. coli*, CL is known to enhance the activity of the glycosyltransferase MurG involved in peptidoglycan biosynthesis [91]. Some correlation (phosphatidic acid > CL > PG) with the activity of dynamin-related Protein 1 (Drp1) has been reported [92]. In addition, CL content plays a critical role since low levels of CL in the membrane of *Pseudomonas putida* allow the tetradecyltrimethylammonium cation (TTAB) to cross the membrane, reach its site of action and kill the cell, as bacteria cannot counteract the fluidizing effect of the detergent [71]. On the contrary, the addition of CL to the culture medium delayed the growth of *P. aeruginosa*, favored asymmetrical growth and enhanced the efficiency of 3′,6-dinonyl NEA **24** [93].

These results shed light on how targeting CL microdomains may be of great interest for developing new antibacterial therapies. Some recent evidence also highlights the role of CL for outer membrane vesicle (OMV) formation. These vesicles could be important as inter-kingdom players. Experimental evidence that bacterial OMVs, by sequestering of cationic peptides, may protect pathogenic yeast against the combined action of antifungal drugs has been reported [94].

#### 2.1.4. Targets and Modes of Action against Gram-Positive Bacteria

We also investigated the mechanism of action against Gram-positive bacteria, *S. aureus* and *Bacillus subtilis* [95]. Time-killing experiments were performed to demonstrate the bactericidal effect induced by 3′,6-dinonyl NEA **24** against *S. aureus* MSSA and MRSA (methicillin susceptible and resistant *S. aureus*, respectively). The displacement of the BODIPY™-TR cadaverine probe bound to lipoteichoic acids (LTA) showed that **24** interacts with the bacterial surface components. The ability of **24** to enhance membrane depolarization and induce membrane permeability was highlighted, using fluorescent probes, DiSC_3_C(5) and propidium iodide, respectively. These effects were observed for both MSSA and MRSA, as well as for *B. subtilis*. The disruption of membrane integrity of the bacterial cell wall was revealed by electronic microscopy, and changes in the localization of lipids from the enriched-septum region and the impairment of the formation of the septum were observed by fluorescence microscopy. This study revealed that **24** interferes with multiple targets, suggesting a low ability of Gram-positive bacteria to acquire resistance to this antibacterial agent.

### 2.2. Recent Reports on AAGs in the Field of Antibacterial Agents

Here, the main reports in the field of antibacterial AAGs, published after the appearance of several review articles since 2016 [7,8,30,31,32], are summarized with an emphasis on AAGs made of an AG core conjugated to an adjuvant or an antibiotic drug of another class (antibiotic hybrids), as recently developed by Schweizer, Zhanel and coworkers.

The grafting of a metal binding site on antimicrobial peptides (AMPs) improves their antibacterial efficiency [96,97,98]. In the search for antibacterial AAGs bearing a metal binding motif, 3′,4′,6-tri2NM NEA (**16**) [22] derivatives functionalized at position 5 through a short spacer by a Zn(II) or Cu(II) chelating group, tris(2-pyridylmethyl)amine (TPA), di(picolyl)amine (DPA) and tetraazacyclotetradecane (Cyclam) were synthesized [99]. NEA-cyclam and Zn(NEA-TPA) derivatives were found to be the most efficient compounds active against clinical MDR strain isolate *Enterobacter aerogenes* EA289, with MICs in the range of 16–4 and 4 μM, respectively, whereas usual antibiotics such as β-lactams and phenicols were inactive and ciprofloxacin was weakly active. NEA-Cyclam and Zn(NEA-TPA) were shown to target and permeabilize the OM of EA289. All NEA conjugates were able to block the efflux of 1,2′-dinaphthylamine in EA289 by acting on the efflux transporter located in the inner membrane [99].

A series of pyrene-NEO (PYR-NEO) conjugates were synthesized and their binding affinity to A-site RNA targets, resistance to AG-deactivating enzymes and antibacterial activity against a wide variety of bacterial strains of clinical relevance were studied [100]. As observed previously with NEA conjugates [35], the conjugation significantly alters the affinities of NEO for bacterial A-site targets. PYR-NEO conjugates exhibited broad-spectrum activity towards Gram-positive bacteria, including improved activity against NEO-resistant methicillin-resistant *S. aureus* (MRSA) strains. The conjugation significantly increased the resistance of NEO to AG-deactivating enzyme modification.

New antibacterial properties of amphiphilic AG conjugates were also recently highlighted. Antimicrobial hybrids that are AAGs have emerged in a promising strategy to combat bacterial resistance as drugs alone and as adjuvants in combination with existing antibiotics. The concepts, advances and challenges of antibiotic hybrids, including AAGs linked to an established antibiotic agent, were reviewed recently [101,102]. Such AAG hybrids can promote the influx of antibiotics through the OM of Gram-negative bacteria via the self-promoted uptake mechanism by displacement of the divalent cations (Ca^2+^ or Mg^2+^) that stabilize LPS [103].

**Figure 9:**
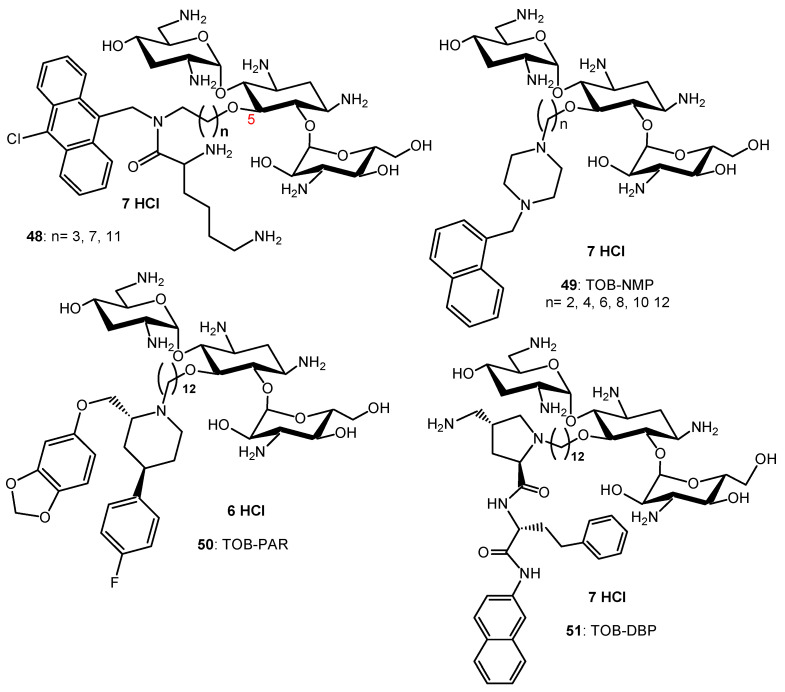
Structures of the tobramycin (TOB) conjugates to lysine **48** [105,106], and to the efflux pump inhibitors (EPIs), 1-(1′-naphthylmethyl)piperazine (NMP) (**49**), paroxetine (PAR) (**50**) and dibasic naphthyl peptide (DBP) (**51**) [107,108].

Lysine-NEO conjugates have been synthesized and exhibited mainly decreased antibacterial activities [104]. Amphiphilic tobramycin (TOB) conjugates to lysine and anthracenyl groups **48** (Figure 9) appeared to be able to sensitize MDR Gram-negative bacteria to antibiotic drugs [105,106].

Combination studies indicate that these amphiphilic TOB-lysine conjugates **48** sensitize Gram-negative bacteria to antibiotics. The most potent derivative (*n* = 11, Figure 9) synergizes rifampicin and minocycline against MDR and extensively drug resistant (XDR) *P. aeruginosa* isolates and enhances efficacy of both antibiotics in the *Galleria mellonella* larvae in vivo infection model [105]. Mode of action studies indicate that the amphiphilic TOB-lysine adjuvants enhance OM cell penetration and affect the proton motive force, which energizes efflux pumps. The TOB-lysine conjugate **48** (*n* = 11) (Figure 9) also potentiates the antibacterial efficacy of eight clinically used antibiotics against wild-type, multidrug-resistant and extensively drug-resistant *P. aeruginosa* isolates from Canadian hospitals [106]. Antibiotics that are synergistic with **48** (*n* = 11) included moxifloxacin (MOX), ciprofloxacin (CIP), erythromycin, chloramphenicol, trimethoprim, novobiocin, linezolid, and fosfomycin. Novobiocin showed the highest synergy.

TOB conjugates to the efflux pump inhibitors (EPIs) 1-(1′-naphthylmethyl)piperazine (NMP) (**49**), paroxetine (PAR) (**50**), and dibasic naphthyl peptide (DBP) (**51**) (Figure 9) also overcome resistance against MDR *P. aeruginosa* isolates by enhancing bacterial OM penetration and reducing efflux [107]. These AAGs enhance the synergy and efficacy of EPIs in combination with tetracycline antibiotics against MDR Gram-negative bacteria, including *P. aeruginosa*. In addition to potentiating tetracycline antibiotics, TOB-EPI conjugates can also suppress resistance development to the tetracycline antibiotic minocycline, thereby providing a strategy to develop more effective adjuvants to rescue tetracycline antibiotics from resistance in MDR Gram-negative bacteria. A strong synergy with MOX, ciprofloxacin (CIP), rifampicin and fosfomycin was also observed against MDR/XDR *P. aeruginosa* [108].

**Figure 10:**
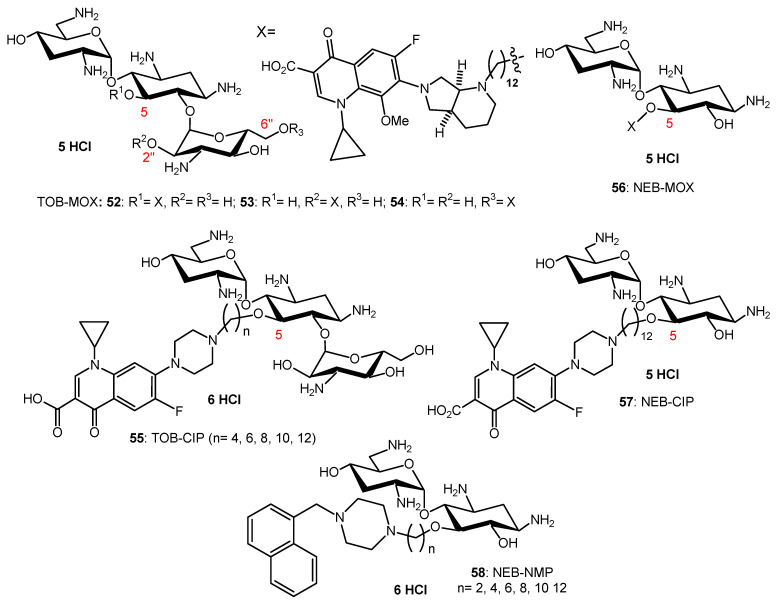
Structures of the TOB (**52**–**54**) and nebramine (NEB) (**56**) conjugates to the fluoroquinolones moxifloxacin (MOX) and ciprofloxacin (CIP) [109,111], respectively **55** and **57**, and, of the NEB conjugates to the efflux pump inhibitor 1-(1′-naphthylmethyl)piperazine (NEB-NMP) **58** [112].

AAG hybrids incorporating a fluoroquinolone were synthesized through conjugation of the MOX and CIP to the TOB core via an aliphatic alkyl linker. Among the TOB-MOX AAGs **52**–**54** (Figure 10) synthesized, the hybrid **52** was shown to enhance OM permeability and reduce efflux by dissipating the proton motive force of the cytoplasmic membrane in *P. aeruginosa* [109]. It protects *Galleria mellonella* larvae from the lethal effects of MDR *P. aeruginosa*. Attempts to select for resistance over a period of 25 days resulted in a two-fold increase in the MIC for the hybrid, whereas MOX or TOB resulted in a 16- and 512-fold increase in MIC. TOB-ciprofloxacin hybrid (TOB-CIP) adjuvants, in which CIP is attached at the TOB 5- (hybrids **55**, Figure 10), 2″- or 6″-position, were shown to rescue the activity of fluoroquinolone antibiotics against MDR and XDR *P. aeruginosa* isolates in vitro and enhance fluoroquinolone efficacy in the *Galleria mellonella* in vivo infection model [110]. Structure–activity studies revealed that the presence of both TOB and CIP, which are separated by a C12 tether, is critical for the function of the adjuvant. Mechanistic studies indicate that the antibacterial modes of CIP are retained, whereas the role of TOB is limited to destabilization of the OM in the hybrid.

In vitro screening of six anticancer drugs for their potential use in antimicrobial therapy suggested the feasibility of repurposing the anticancer drug mitomycin C against MDR Gram-negative bacteria [111]. In combination with the TOB-CIP hybrid **55** (*n* = 12, Figure 10), the antibacterial activity of mitomycin C was enhanced against MDR clinical isolates of *P. aeruginosa*, *A. baumannii*, *E. coli*, *K. pneumoniae* and *Enterobacter cloacae*. Synergy was inherent to TOB-CIP and neither TOB nor CIP individually synergized with mitomycin C. The TOB-based hybrid adjuvants able to potentiate multiple classes of legacy antibiotics against various MDR Gram-negative bacteria were modified by replacing the TOB domain with the smaller pseudo-disaccharide NEB through selective cleavage of the α-D-glucopyranosyl linkage of TOB **8**, which is an NEA (**7**) analogue lacking the 3′-hydroxyl function (Figure 2 and Figure 10) [112]. The hybrid AAGs NEB-moxifloxacin (NEB-MOX) **56**, NEB-ciprofloxacin (NEB-CIP) **57** and NEB-1-(1′-naphthylmethyl)piperazine (NEB-NMP) **58** were synthesized (Figure 10). Potent synergism was found for combinations of NEB-based hybrid adjuvants with multiple classes of antibiotics, including fluoroquinolones (MOX and CIP), tetracyclines (minocycline) and rifamycins (rifampicin) against both wild-type and MDR *P. aeruginosa* clinical isolates. The combination of the optimized NEB-CIP hybrid **56** (Figure 10) and rifampicin protects *G. mellonella* larvae from the lethal effects of extensively drug-resistant (XDR) *P. aeruginosa*. Mechanistic evaluation of NEB-based hybrid adjuvants revealed that the hybrids affect the OM and IM of wild-type *P. aeruginosa* PAO1.

TOB-polymyxin B-3 hybrids showed potent activity against carbapenem-resistant as well as MDR or extensively drug-resistant (XDR) *P. aeruginosa* clinical isolates [113]. The most potent hybrid incorporating a C12 spacer was able to synergize with currently used antibiotics against wild-type and MDR/XDR *P. aeruginosa* and *Acinetobacter baumannii*.

More recently, TOB-rifampicin conjugates were also shown to break the intrinsic resistance in *P. aeruginosa*, due to chromosomally encoded low OM permeability and constitutively over-expressed efflux pumps, and sensitize MDR and XDR *P. aeruginosa* to doxycycline and chloramphenicol in vitro and in vivo [114]. Tetracyclines and chloramphenicol are model compounds for bacteriostatic effects, but when combined with TOB-rifampicin adjuvants, their effects became bactericidal at sub MIC levels. Potentiation of tetracyclines was found to be correlated with SAR of this class of drugs and consistent with OM permeabilization and efflux pump inhibition.

**Figure 11:**
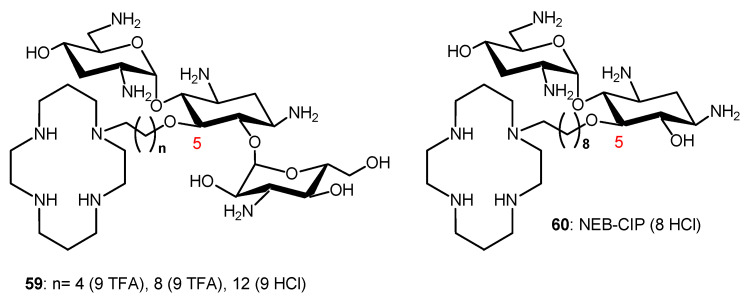
Structures of the TOB-cyclam and NEB-cyclam hybrids **59** [115] and **60** [116].

Conjugation of TOB and cyclam to give **59** (Figure 11) also abrogates the ribosomal effects of TOB but confers a potent adjuvant property that restores the full antibiotic activity of aztreonam and meropenem against carbapenem-resistant *P. aeruginosa* [115]. Therapeutic levels of susceptibility were attained in several MDR clinical isolates, and time-kill assays revealed a synergistic dose-dependent pharmacodynamic relationship. A triple combination of the adjuvant with ceftazidime/avibactam (approved), aztreonam/avibactam (phase III) and meropenem/avibactam enhances the efficacies of β-lactam/beta-lactamase inhibitors against recalcitrant strains, suggesting rapid access of the combination to their periplasmic targets. The adjuvants, and their combinations, were non-hemolytic and non-cytotoxic, and preliminary in vivo evaluation of **59** (*n* = 8) in *G. mellonella* suggested therapeutic potential for the double and triple combinations. The TOB–cyclam conjugate **59** (*n* = 8) mitigates the effect of OprD/OprF porin loss in *P. aeruginosa* and potentiates β-lactam/β-lactamase inhibitors against carbapenem-resistant clinical isolates, highlighting the complexity of resistance to β-lactam antibiotics.

The NEB-cyclam conjugate **60** (Figure 11) was shown to potentiate β-lactam antibiotics, as well as other antibiotic drugs, against *P. aeruginosa* in vitro [116]. This adjuvant is able to synergize with β-lactams aztreonam and ceftazidime against MDR and extremely drug-resistant clinical isolates through a hypothesized mechanism of OM permeabilization.

AGs were also modified for obtaining antibacterial amphiphilic nanoparticles. The brain neurotransmitter dopamine has been polymerized under aerobic conditions to produce polydopamine (PDA). Recently, self-polymerized PDA nanoparticles were tethered to AGs (gentamicin, KANA and NEO) [117]. These nanoconjugates were evaluated for their antimicrobial potency against various bacterial strains, including resistant ones, and their cytocompatibility. Of the three nanoconjugates (PDA-gentamicin, PDA-KANA and PDA-NEO), the PDA-KANA (PDA-K) nanoconjugate exhibited the highest activity against potent pathogens, the least toxicity to human embryonic kidney (HEK 293) cells and intense toxic effects to human glioblastoma (U87) cells.

### 2.3. AAG Positioning as Potential Antibacterial Drug Candidates, Toxicity

**Figure 12:**
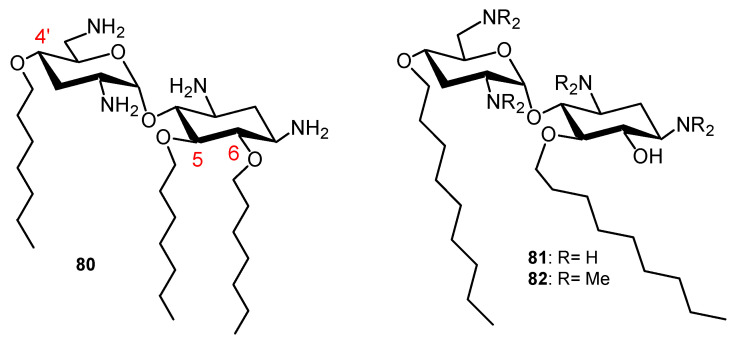
Structures of the synthesized broad-spectrum antibacterial 4′,5,6-tri- and 4′,5-di-alkylated NEB derivatives [37,38].

Dialkyl and trialkyl NEA derivatives have broad-spectrum antibacterial activity against susceptible and resistant Gram-positive and Gram-negative bacteria and their activity is related to favorable lipophilicity windows. Fridman and coworkers identified one amphiphilic 4′,5,6-triheptyl NEB derivative **80** (Figure 12) that is active against susceptible and resistant Gram-positive and Gram-negative bacteria (MRSA, *E. coli*, *K. Pneumonia, P. aeruginosa* strains, etc.) [37]. The corresponding trihexyl and trioctyl derivatives were found to be active against Gram-positive bacteria and inactive or weakly active against Gram-negative bacteria. As a consequence, the antibacterial Gram-positive and Gram-negative activity of amphiphilic NEB derivatives corresponds to a lipophilicity window that is much narrower than the windows delineated in the NEA series for trialkyl and dialkyl derivatives. This result is probably related to the higher lipophilicity of the NEB derivatives in comparison to the corresponding 3′,4′,6-trialkyl NEA derivatives [34,35].

A chemical way to increase the antimicrobial activity of the anti-Gram-positive and anti-Gram-negative NEB, TOB and PARO AAGs was reported in 2015 [38]. AAGs acting against a panel of Gram-positive and Gram-negative bacteria were obtained by di-*N*-methylation of all amine functions of the corresponding AAGs; for example, the 4′,5-dinonyl NEB derivative **81** was methylated to give the tetra-*N*-dimethyl derivative **82**. However, strong hemolytic effects of the *N*-dimethyl NEB, TOB and PARO derivatives were observed and were related to the strong increase in lipophilicity in comparison to the parent non-*N*-dimethylated AAGs (an increase in the calculated logP by about a three orders of magnitude). Cetrimonium and gramicidin D, which are both in antibacterial topical clinical use, appeared to be significantly more hemolytic than all studied antibacterial AAGs. These results suggest a possible means of development of potent and broad-spectrum antibacterial membrane-disrupting AAGs for the treatment of persistent topical infections [38].

In order to evaluate the immediate toxicity of antibacterial AAGs, we performed a toxicity study in SKH1 male mice with AAGs **24** and **26** delivered in aqueous solution at concentrations higher than 5 µM, intravenously, intraperitoneally and subcutaneously in 100 µL injections twice a day. This study revealed immediate toxic effects (tail necrosis or swelling in the area of the injections and high mean body weight decrease; unpublished results obtained in collaboration with the preclinical Contract Research Organization (CRO), VOXCAN, Marcy l’Étoile, France). Such effects could be related, at least in part, to cellular membrane effects and to a low solubility in saline and buffered aqueous solutions. These results show that antibacterial AAGs have to be formulated to be delivered in vivo at high concentrations, for example in liposomes like the anionic and amphiphilic antifungal drug amphothericin B, which self-aggregates and is highly toxic [118]. Such formulations should allow the reduction of the concentration-dependent local toxic effects and the release of the AAGs at an efficient concentration.

The antibacterial potential of AAGs in antibiotherapy as adjuvants to antibiotic drugs also suggests their potential use at lower concentrations, rather than alone as antibiotic drugs. Recently, other adjuvants that are membrane-affecting small molecules were shown to be able to suppress COL resistance by abolishing or reducing the extent of lipid A modification induced by the *mcr-1* gene and pmrAB system in *E. coli* [119,120]. AAGs **24**, **26** and **27** are active against clinical COL-resistant *P. aeruginosa* strains (MICs = 28 µg/mL) and they cooperatively bind to LPS, increasing the OM permeability [42]. The much weaker and slower increase of MIC, in comparison to the fluoroquinolone CIP, of the 3′,6-dinonyl (**24**) and 3′,6-di2NP (**26**) NEA derivatives observed against *P. aeruginosa* illustrates the difficulty of resistance emergence to AAGs. These results suggest that **24** and/or **26** could be used at low concentrations as adjuvants, to reduce resistance to COL.

Another application of antibacterial AAGs could be found in their development as materials useful for antibacterial and anti-biofilm coating of surfaces, for example of catheters. Since trialkyl NEA derivatives are broad-spectrum antibacterial agents, their covalent attachment by one of the three alkyl groups to insoluble crosslinked polymers could produce interesting antibacterial materials.

## 3. Other Biological Activities of AAGs

Since the antibacterial activity of AAGs is related to narrow windows of lipophilicity and their molecular shape, it should be possible to discriminate different targets in adjusting, for example, the AAG lipophilicity and/or the corresponding hydrophilic/hydrophobic balance for different biological and medicinal applications.

### 3.1. Recent Advances in the Field of Antifungal AAGs

**Figure 13:**
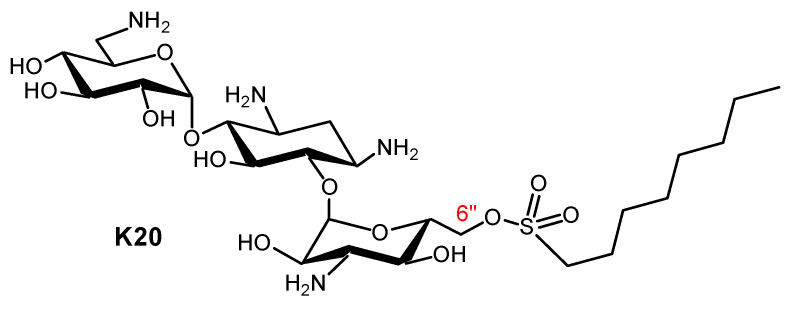
Structure of the amphiphilic aminoglycoside K20, capable of inhibiting many fungal species such as *Fusarium graminearum*, the causal agent wheat *Fusarium* head blight (FHB) [125,126,127,128].

Recent years saw the expansion of AAGs in the search for new antifungal agents and probes for related mechanistic studies. The corresponding advances were reviewed in 2020 [121]. AAGs were shown to disrupt fungal cell membranes. As an example, the first antifungal non-antibacterial AAGs identified were KAN derivatives. Their development, through the delineation of structure–activity relationships, led to the novel antifungal agent K20 (Figure 13), which is capable of inhibiting many fungal species such as *Fusarium graminearum*, the causal agent in wheat *Fusarium* head blight (FHB) [122,123,124,125,126,127,128]. Plasma membrane permeabilization is probably the principal antifungal mechanism of action of AAGs, leading to the suggestion that they mechanistically and structurally comprise a novel class of antifungal agents [121,124,127].

Numerous antifungal AAG derivatives of KANA and KANB, NEB, trehalose, TOB and NEO were identified as antifungal agents or fluorogenic derivatives used as probes for related mechanistic studies. Recently, in a collaboration with D. Aldebert and M. Cornet (TIMC-IMAG/University Grenoble Alpes), we also identified some interesting antifungal antibacterial amphiphilic NEA and neosamine derivatives (unpublished results).

### 3.2. AAG Vehicles for Nucleic Acids, Effects on DNA or RNA

#### 3.2.1. Gene and siRNA Delivery

AAGs have also received interest in relation to different medicinal applications, especially for intracellular delivery of active agents, for example, nucleic acids or analogues, non-covalently or covalently bound to AAGs or AGs. The synthesis and development of gene delivery vehicles was recently reviewed [129]. Here, we mainly illustrate the precursor works of J.M. Lehn, P. Lehn and coworkers.

**Figure 14:**
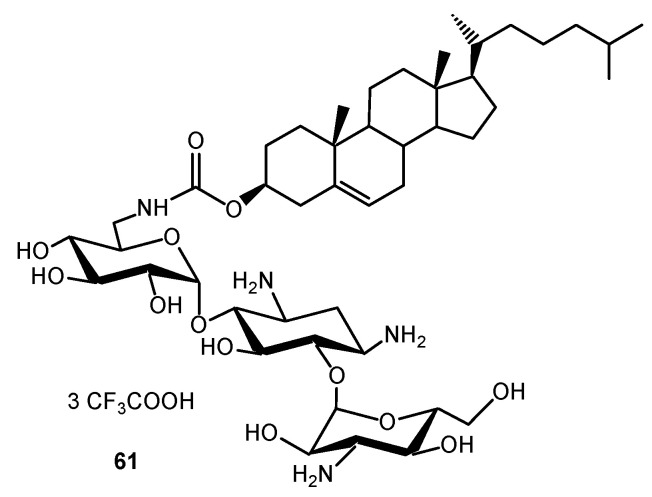
Structure of one of the kanamycin (KANA)-cholesterol conjugates, **61**, developed for gene transfection [130].

Amphiphilic TOB, KANA, PARO, NEO and NEA derivatives were demonstrated to be efficient intracellular delivery vehicles for biologically relevant molecules such as genes [130,131,132,133,134] and small interfering RNA (siRNA) [135]. The transfection potential of KANA and of its tri-guanidinylated derivative, conjugated to cholesterol through the formation of a carbamate group, was explored [130,131,132]. The KANA conjugate **61** (Figure 14) was highly efficient for gene transfection into a variety of mammalian cell lines when used either alone or as a liposomal formulation with the neutral phospholipid dioleoylphosphatidylethanolamine (DOPE). The polyguanidinylated derivative mediated in vitro gene transfection. In addition, the colloidally stable KANA-cholesterol conjugate/DOPE lipoplexes were found to be efficient for in vivo gene transfection into mouse airways. AAGs consisting of cholesteryl or dioleyl moieties linked via various spacers to PARO or NEO headgroups are also efficient for gene transfection both in vitro and into mouse airways in vivo, and the physico-chemical properties of their DNA lipocomplexes were investigated in order to delineate structure–activity relationships [130].

**Figure 15:**
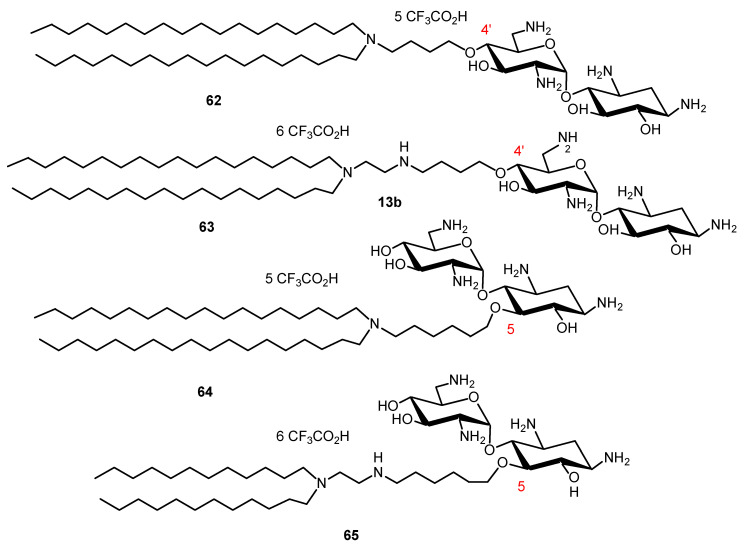
Structures of the most efficient NEA-based vectors for gene transfection [134].

NEA derivatives **62**–**65**, bearing long dialkyl chains, one or two NEA headgroups and four to ten protonatable amine functions, were also prepared for gene transfection through the selective alkylation of the 4′- or 5-hydroxyl function in ring I and ring II of NEA, respectively (Figure 14) [134]. The transfection activity of the twelve derivatives synthesized was investigated in vitro in gene transfection experiments, using several mammalian cell lines. The most efficient derivative, **63** (Figure 15), carries two octadecyl chains linked to a diaminoethyl spacer attached at the 4′-position of the NEA core. Both the presence of that spacer and such an attachment position, i.e., at an extremity of the NEA core, appear to play a role in the increase of the gene transfection efficiency.

**Figure 16:**
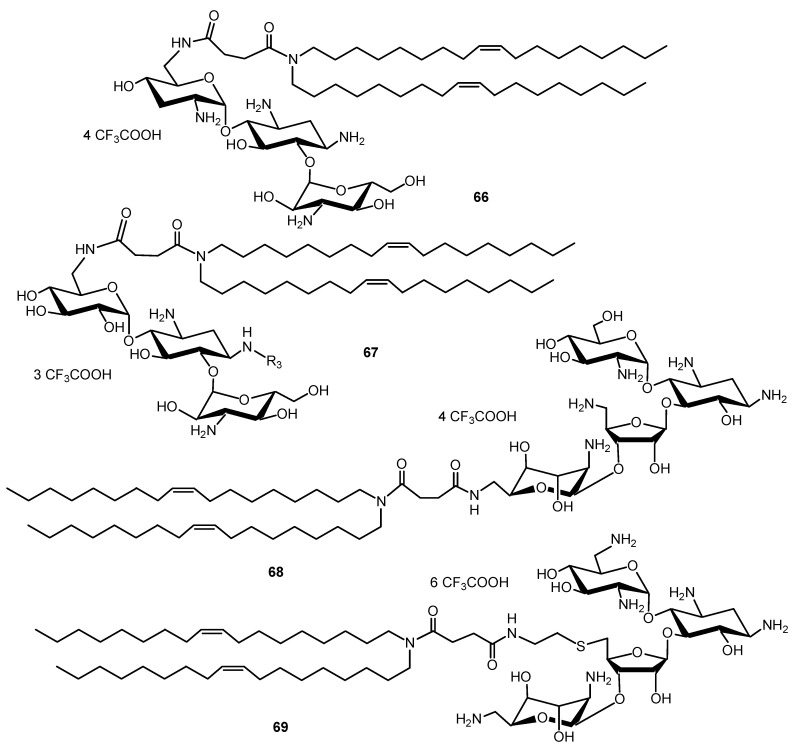
Structures of the AAGs developed for small interfering RNA (siRNA) delivery made of the TOB, KANA, PARO and NEO cores, respectively, linked to two dioleyl chains by a succinyl spacer [135].

Chemically synthesized small interfering RNAs (siRNAs) can be used to induce specific and reversible gene expression silencing in mammalian cells. Cationic lipids commonly used for DNA transfection have also been used for siRNA transfection. To deliver siRNA into cells, two dioleyl chains were grafted through a succinyl spacer to the TOB, KANA, PARO and NEO cores to give AAGs **66**, **67**, **68** and **69**, respectively (Figure 16) [135]. The most active AAG/siRNA complexes for gene silencing were the PARO and NEO derivatives **68** and **69**, which were characterized as small particles exhibiting lamellar microdomains, corresponding to siRNA sandwiched between the lipid bilayers.

More recently, in the search for gene delivery vehicles with inherent antibacterial properties, NEO AAGs **1**, PARO **2** and NEA **6** were conjugated to the tetramino-tetrahexyloxycalix [4]arene scaffold [136]. The three synthesized conjugates exhibited greater DNA binding ability than the gold standard transfectant 25 kDa *b*PEI, as well as a striking DNA packing ability. An antibacterial effect of the NEO and PARO AAGs and their DNA lipoplexes was observed against *E. coli*.

#### 3.2.2. Peptide (Polyamide) Nucleic Acid (PNA)-AG Conjugates to Target RNA

In the antisense approach developed to target, for example, messager RNA sequences by hybridization and to inhibit the corresponding translation, conjugates of AGs (NEO, NEA, PARA, ribostamycin and methyl neobiosamine) to oligo-2′-deoxyribonucleotides (ODNs) were synthesized in order to allow the ODN cellular uptake [137,138,139,140,141,142]. However, in the resulting conjugates, the strong binding of AGs to the ODN through intramolecular charge–charge interaction between the protonated AG core and the phosphodiester backbone can disturb the selective binding to the RNA target.

Peptide nucleic acid oligomers (PNAs) are a class of antisense DNA analogues devoid of charges under physiological conditions, first synthesized by Buchardt, Nielsen and coworkers in 1991 [143]. Their design, based upon a repeating *N*-(2-aminoethyl)glycine polyamide backbone to which nucleobases are attached through a methylene carbonyl linkage to the α-amino group, allows the perfect H-bonding to the RNA and DNA complementary sequences. PNAs are chemically stable and show superior resistance to nucleases and proteases than nucleic acids. However, their low solubility in water and their poor cellular uptake hampered their development in the antisense approach. PNAs can be used in an antibacterial approach, consisting in specifically targeting any single pathogen. For example, potent antibacterial antisense peptide–peptide nucleic acid conjugates were developed against *P. aeruginosa* [144]. A recent review highlighted the potential of gene-specific oligonucleotides and PNAs as antibacterial agents in order to broaden the range of potential targets to any gene with a known sequence in any bacterium [145].

AGs are attractive cationic vehicles to conjugate to PNAs. The resulting AAG conjugates should be more soluble in water than the PNAs, and the conjugation could allow an efficient cellular uptake of the PNAs. Therefore, chemical methods for the conjugation of PNAs to AGs have been developed [142,146,147,148,149,150]. We conjugated the NEA core with a 16-mer PNA targeting HIV-1 TAR RNA (anti-TAR PNA) [146]. In the resulting conjugate **73** (Figure 16), the presence of the NEA core not only strongly increased the solubility in water and enhanced the cellular uptake of PNA, it also conferred a unique metal ion-independent, target-specific RNA cleavage property to the conjugate [146,148].

**Figure 17:**
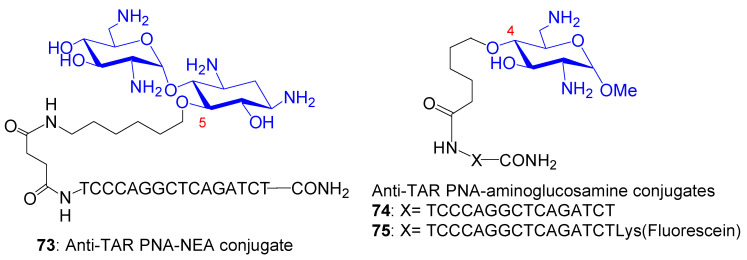
Structures of the anti-HIV (anti-TAR RNA) PNA conjugates to NEA [145,149] and to 6-amino-6-deoxy-1-methylglucosamine (1-methyl neosamine) [151].

We also conjugated the 6-amino-6-deoxy-1-methylglucosamine (1-methyl neosamine) core corresponding to ring II of NEA, to a 16-mer PNA targeting HIV-1 TAR RNA [151]. The resulting conjugate **74** (Figure 17) was stable under acidic conditions and displayed very high target specificity in vitro and strongly inhibited Tat-mediated transactivation of HIV-1 LTR transcription in a cell culture system. The fluorescent derivative **75** was efficiently taken up by the human cells and well distributed in both the cytosol and nucleus without endosomal entrapment because co-treatment with endosome-disrupting agent had no effect on its cellular distribution.

A strategy consisting of recognizing and selectively targeting RNA duplex sequences (ds) with chemically modified dsRNA-binding PNAs (dbPNAs), incorporating thiopseudoisocytosine (L) and guanidine-modified 5-methyl cytosine (Q) residues at physiologically relevant conditions, was developed to facilitate the sequence-specific recognition of Watson−Crick G-C and C-G pairs [152]. A short 10-mer dbPNA targeting a highly conserved panhandle duplex structure of influenza A virion, not accessible to traditional antisense DNA or RNA with a similar length, was conjugated to the NEA core to enhance the cellular uptake [153]. It was shown to inhibit innate immune receptor RIG-I binding to panhandle structure and thus RIG-I ATPase activity. These findings provide the foundation for developing novel dbPNAs for the detection of influenza viral RNAs and therapeutics with optimal antiviral and immunomodulatory activities. An antisense PNA was also conjugated to the NEA core to enter cells and was used to target and probe the tau pre-mRNA exon 10 5′-splice hairpin structure through strand invasion [154].

#### 3.2.3. Some Particular Effects of AAGs on DNA and RNA

The study of 6″-substituted variants of TOB, derivatives with C-12 or C-14 linear alkyl substituents, revealed a potent inhibition of reverse transcription in vitro, which was related to nucleic acid binding with high affinity and the formation of high-molecular weight complexes [155]. Stable complex formation was observed with DNA or RNA in single- or double-stranded form, suggesting that the formation of micelles and/or vesicles with surface-bound nucleic acids may be a useful tool to localize nucleic acids to surfaces or deliver nucleic acids to cells or organelles.

**Figure 18:**
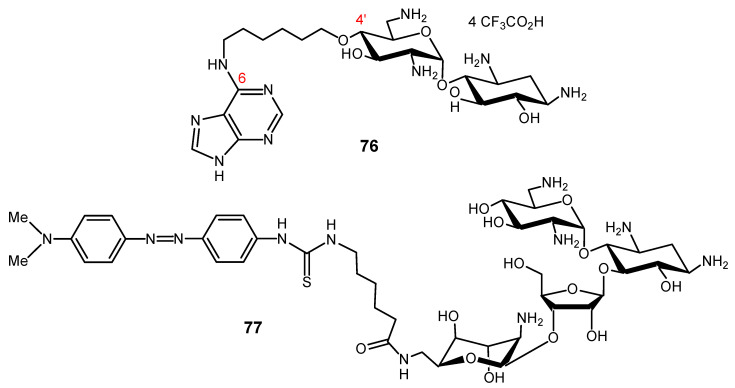
Structure of the most efficient DNA-cleaving AAG identified, **76,** at abasic sites [157], and of the amphiphilic azobenzene-NEO conjugate **77** forming nanostructures [158].

Abasic sites are probably the most common lesions in DNA, resulting from the hydrolytic cleavage of glycosidic bonds that can occur spontaneously and through DNA alkylation, especially at *N*-7 of purine [156]. The Nobel Prize in Chemistry for 2015 was awarded to T. Lindahl, P. Modrich and A. Sancar for mapping at a molecular level how cells repair damaged DNA in order to preserve genetic information. Compounds able to specifically bind and react at abasic sites have attracted much attention for therapeutic and diagnostic purposes. We studied the efficient cleavage activity of AG antibiotic drugs and of some AAGs at abasic sites, introduced either by depurination in a plasmidic DNA or site-specifically in a synthetic oligonucleotide [157]. NEO was found to be the most efficient cleaving AG drug, followed by the AAG **76** (Figure 18) resulting from the conjugation of the NEA core to the nucleic base adenine (EC_50_ = 0.09 and 0.15 µM, respectively, and EC_50_(NEA) = 0.9 µM).

### 3.3. AAGs as Intracellular Delivery Vectors of Drugs

The cationic amphiphilic stimuli-responsive azobenzene-NEO conjugate **77** (Figure 18) was synthesized and its self-assembly in aqueous solutions into nanostructures, in which eosin and aspirin were successfully encapsulated, was demonstrated [158]. The ability of the resulting nanostructures to act as drug carriers, in a UV- and visible light-mediated release pattern or through azoreductase-mediated cleavage of the azo moiety, was also investigated. Stimuli responsiveness of nanostructures and their on/off-switch-like behavior appeared to be of interest, in relation to their use as controlled drug delivery systems and in other biomedical applications, such as colon-specific delivery and gene delivery. Amphiphilic PARO-derived nanoparticles were also prepared for drug delivery from mPEG-PAE (methoxy-terminated poly(ethylene glycol)-poly(aminoether)) nanoparticles [159]. They were able to carry significant amounts of the anticancer drug doxorubicin, and cell-based studies indicated that the nanoparticles loaded with doxorubicin were able to induce a significant loss in the viability of cancer cells.

### 3.4. A New Target of AAGs

**Figure 19:**
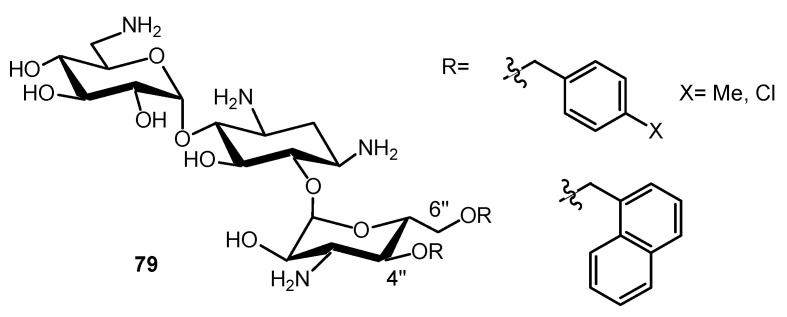
Structures of AAGs that are non-bactericidal and non-toxic or moderately toxic to mammalian HeLa cells, which are connexin hemichannel (HC) inhibitors [160].

Connexin hemichannels (HCs) from adjacent cells form gap junctional channels to mediate cell-to-cell communication. Abnormal opening of “free” undocked HCs can produce cell damage and participate in the mechanism of disorders such as cardiac infarction, stroke, deafness, skin diseases and cataracts. Therefore, inhibitors of connexin HCs have great pharmacological potential. AGs have been recently identified as connexin HC inhibitors, but their antibiotic effect is an issue for the treatment of disorders where infections do not play a role [160]. Several AAGs without antibiotic effect were synthesized and tested for their inhibition against connexin HCs, using a newly developed cell-based bacterial growth complementation assay. Several leads, **79** (Figure 19), that are non-bactericidal and non-toxic or moderately toxic to mammalian HeLa cells, with superior potency than the parent compound, KANA, were identified.

## 4. Discussion and Conclusions

AAGs have been found to have many potential applications of biological and medicinal interest. Several interesting key targets were identified for antibacterial AAGs—ribosomal RNA (a target of AAGs carrying small lipophilic groups) [9,10], as well as LPS [25,42,89] and cardiolipin [89,90,93] in membranes of Gram-negative bacteria and lipoteichoic acids in membranes of Gram-positive bacteria [95]. The presence of these different bacterial targets allows for broad-spectrum antibacterial activity and limits the emergence of resistance to antibiotic AAGs. It could be a limitation in the bacterial/mammalian cell selectivity leading to toxic effects, since cardiolipin is present in mammalian cell membranes. The selectivity of AAGs for bacterial membranes should be improved through mechanistic studies and the careful design of the active species, for example, in order to selectively target LPS or cardiolipin clusters, especially LPS, which is a unique component of bacterial membranes.

We showed that the broad-spectrum antibacterial activity of AAGs is dependent on their lipophilicity, corresponding to narrow windows of clogP (or clogD) [34,35]. Several types of AAGs may be defined, for example, from the composition of their lipophilic part. A first type corresponds to antibacterial and/or antifungal AAGs carrying one or more lipophilic groups (alkyl, arylalkyl) including only carbon and hydrogen atoms, like broad-spectrum antibacterial NEA and PARA derivatives [5,34,35,36]. Such AAGs target bacterial and/or fungal membranes. The identification of non-antibacterial antifungal AAGs targeting fungal membranes demonstrates that the main targets of AAGs can be discriminated [121,122,123,124,125,126,127]. This selectivity could be found in the difference between monoalkylated and di- or tri-alkylated derivatives of similar lipophilicities.

A second type of AAGs corresponds to AG conjugates in which the lipophilic part incorporates heteroatoms (mainly O and N). In a promising strategy to combat bacterial resistance, antibacterial AAGs have also emerged as adjuvants in combination with existing antibiotics. AG conjugates to an efflux pump inhibitor or to an antibiotic drug of another class (antibiotic hybrids) can promote, as adjuvants, the uptake of antibiotics through the OM of Gram-negative bacteria via the self-promoted uptake mechanism by displacement of the divalent cations (Ca^2+^ or Mg^2+^), which stabilize LPS [101,102,103,104,105,106,107,108,109,110,111,112,113,114,115,116]. The high affinity of strongly lipophilic AAGs of the first and second types for nucleic acids was used to develop efficient non-antibacterial vehicles for nucleic acid uptake in mammalian cells [129,130,131,132,133,134,135]. AAGs have also been employed for drug delivery [158,159] and their cationic characters should be exploited to deliver anionic drugs. More recently, weakly lipophilic monoalkyl AAGs were demonstrated to be non-antibacterial inhibitors of connexin hemichannels [160].

Altogether, these results suggest that it should be possible to discriminate different anionic targets through a fine adjustment of the lipophilicity, of the number of positive charges present at physiological conditions, of the hydrophilicity/hydrophobicity balance and the corresponding molecular shape of AAGs. In our opinion, the number of charges should be limited in order to increase the selectivity, as well as the lipophilicity, which increased the cytotoxicity in the NEA series. The detailed understanding of the mechanisms of the antibacterial and antifungal effects, as well as the internalization in mammalian cells, of AAGs having different lipophilicities, and of their lipoplexes, should contribute strongly to a structural design that can improve their activity and their selectivity. There is no doubt that they will be used as efficient tools in many future biological, pharmacological and medicinal studies.

## Figures and Tables

**Table 1:** Minimum inhibitory concentrations (MICs) of the NEA derivatives **24**, **26**, **39** and of NEO **1** and NEA **6**, representative aminoglycosides (AGs) against susceptible and resistant *Staphylococcus aureus* and *Pseudomonas aeruginosa* strains [35]. MRSA: methicillin-resistant *S. aureus*.

AGs	Lipophilicity Expressed as clogP	MIC µg/mL						
		*S. aureus*			*P. aeruginosa*			
		ATCC 25923	SA-1 Pump NorA	ATCC 33592 HA-MRSA	ATCC 27853	Psa. FO3 ^a^	PA22 ^b^	PA406 ^c^
NEO **1**	−29.9	1–2	0.5–1	>128	64	128	32–64	2–4
NEA **6**	−19.4	16–32	8	>128	>128	>128	>128	64
3′,6-diNn **24**	−11.9	1	1	2–4	2–4	4–8	4	2–4
3′,6-di2NP **26**	−11.4	2	2	2	8-16	16	16	2–4
3′,6-diOc **39**	−12.7	1	1	2	2	8	8	2

^a^: Psa.F03 AAC6′-IIA; ^b^: surexp MexXY; ^c^: PAO509.5 ∆triABC.

**Table 2:** Viability (%) of murine J774 macrophages determined using the MTT assay in the presence of 10 and 30 µM of the NEA derivatives **24**, **26** and **39** in comparison to NEO **1** and NEA **6**, representative AGs; the numbers of independent experiments are mentioned after the viability values in brackets [35].

AAG	Lipophilicity Expressed as clogP	Viability %	
		10 µM	30 µM
NEO **1**	−29.9	87.3 (10)	69.8 (2)
NEA **6**	−19.4	94.8 (9)	84.4 (2)
3′,6-diNn **24**	−11.9	86.7 (9)	67.4 (3)
3′,6-di2NP **26**	−11.4	91.1 (13)	89.5 (2)
3′,6-diOc **39**	−12.7	91.3 (4)	65.1 (3)

## Abbreviations

AAG	Amphiphilic aminoglycoside
AG	Aminoglycoside
Bu	*n*-butyl
CIP	Ciprofloxacin
CL	Cardiolipin
clogP	Calculated log(octanol/water partition coefficient)
COL	Colistin
DiMOc	3,7-(dimethyl)octyl
DBP	Dibasic naphthyl peptide
De	Decyl
EPI	Efflux pump inhibitor
Hx	*n*-hexyl
Hp	*n*-heptyl
IM	Inner membrane
KANA	Kanamycin A **3**
KANB	Kanamycin B **4**
LPS	Lipopolysaccharides
MDR	Multidrug-resistant
MIC	Minimum inhibitory concentration
MOX	Moxifloxacin
MRSA	Methicillin resistant *S. aureus*
2NB	2-naphthylbutyl
NEA	Neamine **6**
NEB	Nebramine **8**
NEO	Neomycin B **1**
2NH	2-naphthylhexyl
2NM	2-naphthylmethyl
NMP	1-(1′-naphthylmethyl)piperazine
Nn	*n*-nonyl
2NP	2-naphthylpropyl
Oc	*n*-octyl
Ocd	*n*-octadecyl (C18)
OM	Outer membrane
PAR	Paroxetine
PARA	Paromamine **7**
PARO	Paromomycin **2**
PDA	Polydopamine
PE	Phosphatidylethanolamine
PG	Phosphatidylglycerol
PMB	*para*-methoxybenzyl
POPE	1-palmitoyl-2-oleoyl-sn-glycero-3-phosphatidylethanolamine
POPG	1-palmitoyl-2-oleoyl-*sn*-glycero-3-phospho-(1′-*rac*-glycerol)
PNA	Peptide (polyamide) nucleic acid
SAR	Structure-activity relationships
TOB	Tobramycin **5**
Tr	Trityl
Ud	*n*-undecyl (C11)
XDR	Extensively drug-resistant
